# Comparative Genomic Analyses Reveal Core-Genome-Wide Genes Under Positive Selection and Major Regulatory Hubs in Outlier Strains of *Pseudomonas aeruginosa*

**DOI:** 10.3389/fmicb.2019.00053

**Published:** 2019-02-06

**Authors:** Utkarsh Sood, Princy Hira, Roshan Kumar, Abhay Bajaj, Desiraju Lakshmi Narsimha Rao, Rup Lal, Mallikarjun Shakarad

**Affiliations:** ^1^Department of Zoology, University of Delhi, New Delhi, India; ^2^PhiXGen Private Limited, Gurugram, India; ^3^Department of Veterinary & Biomedical Sciences, South Dakota State University, Brookings, SD, United States; ^4^National Centre for Microbial Resource, National Centre for Cell Science, Pune, India; ^5^ICAR-Indian Institute of Soil Science, Bhopal, India

**Keywords:** *Pseudomonas aeruginosa*, outliers, environmental genomics, drug targets, positive selection

## Abstract

Genomic information for outlier strains of *Pseudomonas aeruginosa* is exiguous when compared with classical strains. We sequenced and constructed the complete genome of an environmental strain CR1 of *P. aeruginosa* and performed the comparative genomic analysis. It clustered with the outlier group, hence we scaled up the analyses to understand the differences in environmental and clinical outlier strains. We identified eight new regions of genomic plasticity and a plasmid pCR1 with a VirB/D4 complex followed by trimeric auto-transporter that can induce virulence phenotype in the genome of strain CR1. Virulence genotype analysis revealed that strain CR1 lacked hemolytic phospholipase C and D, three genes for LPS biosynthesis and had reduced antibiotic resistance genes when compared with clinical strains. Genes belonging to proteases, bacterial exporters and DNA stabilization were found to be under strong positive selection, thus facilitating pathogenicity and survival of the outliers. The outliers had the complete operon for the production of vibrioferrin, a siderophore present in plant growth promoting bacteria. The competence to acquire multidrug resistance and new virulence factors makes these strains a potential threat. However, we identified major regulatory hubs that can be used as drug targets against both the classical and outlier groups.

## Introduction

*Pseudomonas aeruginosa* is one of the major opportunistic pathogens that has been isolated from a wide range of ecological niches including soil, water and clinical samples (Stover et al., 2000). This opportunistic pathogen is prototypical “multidrug-resistant” (MDR) with intrinsically advanced antibiotic resistance gene clusters (Lambert, 2002; Livermore, 2002). Some of the strains of *P. aeruginosa* have been associated with serious illnesses mainly imparting nosocomial infections (ventilator-associated pneumonia) and various sepsis syndromes (Planquette et al., 2013; Philippart et al., 2015). The pathogenicity of *P. aeruginosa* is attributed to the presence of prominent virulence factors that consist of proteases, pili, flagella, quorum sensing proteins, exotoxin A and type III secretion system (T3SS) (Lyczak et al., 2000). Amongst these, the T3SS- also called injectosome is responsible for the injection of toxins into the eukaryotic host cell and thus majorly determines the degree of pathogenicity (Cornelis, 2006). Therefore, strains lacking T3SS were thought to be less pathogenic due to the absence of injectosome machinery and its effector toxins (ExoS, ExoT, ExoU, and ExoY) that play a prominent role in acute as well as chronic infections (Hauser, 2009). These T3SS deficient strains are termed as outlier strains and they form a distinct phylogenetic clade represented by strain PA7 (Roy et al., 2010); and were shown to have diverged early from the classical strains of *P. aeruginosa* (Gomila et al., 2015). The outliers counteract T3SS deficiency by adopting novel virulence factors and attaining multidrug resistance, thus making them difficult to treat using antibiotics (Roy et al., 2010; Huber et al., 2016). An outlier clinical isolate of *P. aeruginosa* strain CLJ1 was reported to induce hemorrhagic pneumonia, mainly due to the presence of a two-partner secreted Exolysin (ExlA) with membrane lytic activity (Elsen et al., 2014). The two genes encoding this lytic toxin are present on a region of genomic plasticity (RGP) that is associated with outlier *P. aeruginosa* (Huber et al., 2016; Reboud et al., 2016). Although strains of *P. aeruginosa* are ubiquitous and there are more than 1500 assemblies present for classical strains, only a handful of draft genomes (*n* = 12) (Boukerb et al., 2015; Kos et al., 2015; Mai-Prochnow et al., 2015; van Belkum et al., 2015) are present in the genome database for outlier strains (either clinical isolates or with unknown origin), with a single complete genome of strain PA7 (Roy et al., 2010).

In this study, a new outlier strain CR1 of *P. aeruginosa*, an isolate from the rhizosphere of chili crop was genomically characterized (Supplementary Table S1). Its complete genome was sequenced and compared with other outliers of clinical and unknown origin. Virulence factors in association with acquired resistance play a significant role in inducing pathogenicity, therefore the genes associated with virulence and antibiotic resistance were analyzed in this environmental isolate. Here, we also report the presence of a plasmid pCR1 from strain CR1 which is not present in the genome of any of the outlier strains. The plasmid had VirB/D4 type IV secretion system in association with trimeric autotransporter adhesin protein having similarity with *yadA* like adhesin that can impart a pathogenic potential to CR1 (Christie, 2001; Linke et al., 2006; Basso et al., 2017). Like other outliers, CR1 lacks T3SS which majorly confers pathogenicity. The probability of these strains to be a pathogen was also assessed based on the number of pathogenic protein families present in the genome. Differences in the core and pan-genes of the two groups were determined. Core genome-wide genes under positive selection were determined for identifying the genes under strong positive selection that can provide advantageous variants to the outlier genomes. The ability of these strains to gain virulence factors and acquired antibiotic resistance require new drug targets to be identified. Therefore, protein–protein interaction on the core genome of both the groups was used to identify major regulators that can be targeted as drug targets.

## Materials and Methods

### Genome Sequencing and Assembly

The *Pseudomonas* strain CR1 used in the study is an isolate from the rhizosphere of chili crop grown with high inputs of chemical fertilizers and pesticides at Guntur, Andhra Pradesh, India (Supplementary Table S1). The DNA was extracted using Cetyl Trimethyl Ammonium Bromide (CTAB) extraction method (Wilson, 1987) and sequenced using Single Molecule Real Time sequencing (SMRT, Pacific Biosciences) at the Quebec Innovation Centre McGill University, Canada. The 16S rRNA sequence homology of the strain revealed its identity as *Pseudomonas aeruginosa.*

A total of 73,250 subreads of average length 10,488 bp were obtained from Pac Bio RS II with 20 kb library (Pacific Biosciences, Menlo Park, CA, United States). The reads were assembled into 2 contigs, using the HGAP2 algorithm (Chin et al., 2013) in SMRT Analysis 2.1 (Pacific Biosciences).

### Selection and Annotation of Genomes for Comparative Analysis

For comparative genomic analysis, complete genomes of 50 *P. aeruginosa* strains (including the complete genome of PA7) were retrieved from the *Pseudomonas* Genome Database (Winsor et al., 2016) (Supplementary Table S2, data accessed on 14.07.2016). Further to increase the resolution, 15 draft genomes reported as the closest neighbor by RAST neighbor score were also included in the analysis. Preliminary analysis revealed that the strain CR1 was a member of the outlier PA7 clade. Therefore, we included all the draft genomes of outlier strains (*n* = 12) of *P. aeruginosa* available till 14.07.2016. The genomes were annotated by RAST server 2.0 (Aziz et al., 2008) and Glimmer-3 (Delcher et al., 2007) was used for predicting genes. The 12 draft genome of outliers and two complete genomes of strain CR1 and PA7 were taken for genomic comparison among outliers. The draft genomes were checked for completeness by analyzing the presence of 107 essential copy genes using the Comprehensive Microbial Resource as a database, where 107 Hidden Markov models (HMMs) of essential copy genes were analyzed in all phylogenetic outlier strains (Dupont et al., 2012).

Further, the 14 outlier strains were annotated for RNAs and tRNAs using RNAmmer (Lagesen et al., 2007) and ARAGORN, (Laslett and Canback, 2004), respectively. The CRISPR elements within the outlier strains were analyzed using CRISPR finder server (Grissa et al., 2007). The Phage genomic content was determined using PHAST (Zhou et al., 2011). The sequence type (ST) was also predicted for the strains clustered in outlier clade using MLST 1.8 tool (Larsen et al., 2012). The whole genome sequence of strain CR1 was deposited to PubMLST server (Jolley et al., 2018) for assigning new ST. The DNA-DNA hybridization values were also calculated to delineate at sub-species level using the Genome to Genome Distance Calculator (GGDC v 2.1) for the outliers (Auch et al., 2010a,b).

### Phylogenomics Analysis

All *Pseudomonas* strains (*n* = 78) were subjected to phylogenetic/phylogenomic analysis using four different methods *viz*. Mummer based Average Nucleotide Identity (ANIm), PhyloPhlAn (Segata et al., 2013), phylogeny based on SNPs within the core genome using Harvest suite (Treangen et al., 2014) and pangenome consensus matrix using get_homologues (Contreras-Moreira and Vinuesa, 2013). The ANIm values were calculated for each pair of genomes using pyani^1^ module based on the Mummer algorithm at default parameters. Thereafter, a dual dendrogram based on ANIm values, was constructed in R (R Development Core Team, 2015). Further, to obtain a consensus tree topology, amino acid sequences predicted by Glimmer-3 were used for phylogenomic reconstruction using PhyloPhlAn. PhyloPhlAn locates 400 ubiquitous bacterial genes by sequence alignment in amino acid space, then builds a tree by concatenating the most discriminative positions in each gene into a single long sequence and applying FastTree (Price et al., 2009), and RaxML (Stamatakis, 2014) to construct phylogenetic tree based on maximum likelihood with 1000 bootstrap replicates. Unrooted maximum likelihood (100 bootstraps) tree of 78 *P. aeruginosa* genomes based on SNPs within the core genome was defined using Parsnp tool of the Harvest suite.

Pangenome matrix was also used to construct the dendrogram for closely related *P. aeruginosa* strains in order to obtain better resolution of phylogenetic clades. The consensus pangenome matrix was generated using the intersection of pan-genes predicted by COG and OMCL algorithms using get_homologues. This matrix was then used to construct a parsimony tree using PARS program from the PHYLIP suite to deduce the phylogeny implied in this matrix.

### Determination of Regions of Genome Plasticity (RGP)

**FIGURE 1:**
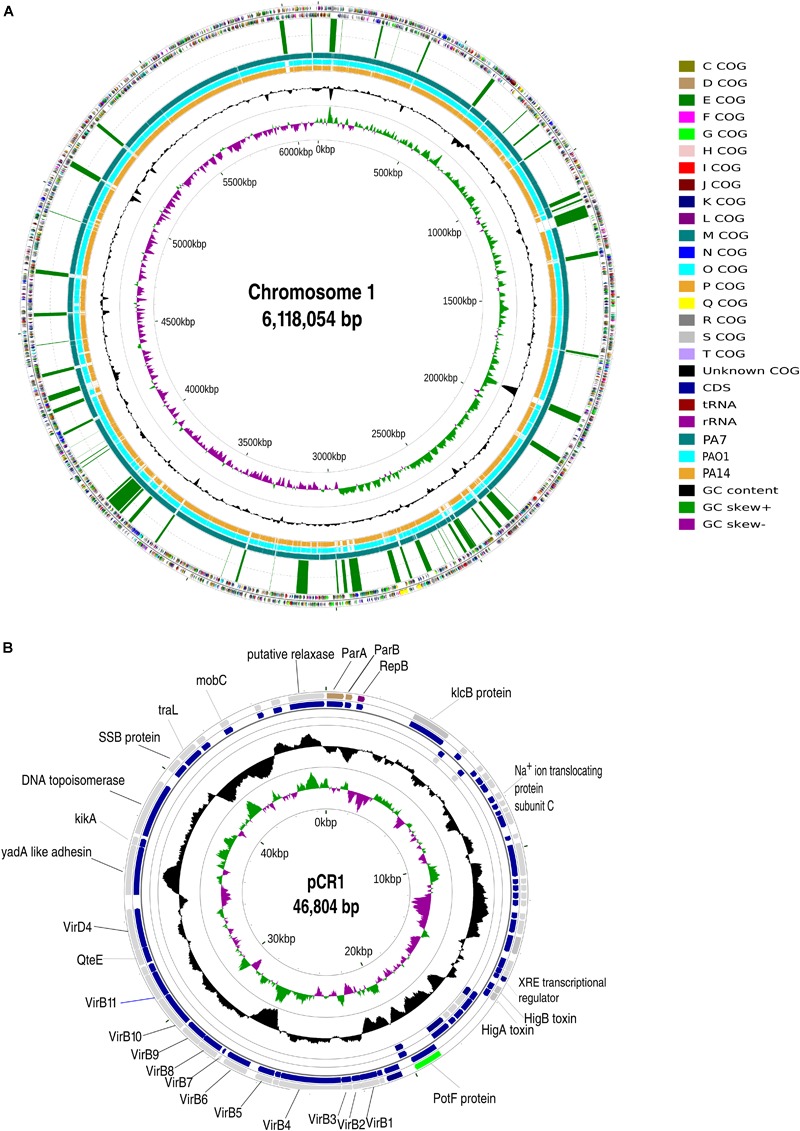
General genomic attributes of *Pseudomonas aeruginosa* CR1. **(A)** Circular map of chromosome 1 of CR1 strain. From outside to inside Ring 1: COG categories of ORFs in the positive strand. Ring 2: COG categories of ORFs in the negative strand. Ring 3: Regions of Genomic plasticity starting from zero bp mark in a clockwise direction. Ring 4: BLASTn alignment (expected threshold = 1*e*-220) between CR1 and previously sequenced outlier PA7 (teal); Ring 5: PAO1 (aqua); Ring 6: UCBPP-PA14 (orange). Rings 7 and 8: GC content and GC skew, respectively. **(B)** Circular map of plasmid pCR1. From outside to inside Rings 1, 2, 3, and 4 depicts COG categories of ORFs of the positive strand, genes on positive strand, genes on negative strand and COG categories of ORFs of negative strand, respectively. Rings 5 and 6 represents GC content and GC skew, respectively.

The strain CR1 was analyzed for regions of genomic plasticity (RGP) using the standard genome subtractive approach on pairwise genome comparisons with reference genomes of PAO1, UCBPP-PA14, and PA7. The criteria to characterize and discriminate RGPs from other features in a genome were based on the following: atypical composition, presence of tRNA- that serves as insertion sites, presence of insertion sequence (IS) elements and direct repeats (DR) flanking RGPs, presence of integrase and transposase (Langille et al., 2010; Che et al., 2014). These RGP were then plotted in genome map using CG view (Grant and Stothard, 2008) in the circular map of the chromosome 1 (Figure 1A).

### Orthologs Under Positive Selection

The orthologs were predicted using OrthoMCL v1.4 using –mode 1. The orthologous groups were then added to POTION v 1.1.3 pipeline (Supplementary File S1) (Hongo et al., 2015). The genes in the orthologous group were detected for recombination signals and removed using PhiPack (Bruen and Bruen, 2005). For all genes tested for positive selection, the false discovery rate (FDR) was controlled using the BY method (Benjamini and Yekutieli, 2001) and the significance level was set to 10%. The rates of synonymous and non-synonymous substitutions were estimated using site-model of the codeml program in the PAML v4.8 package (Yang, 2007). trimAl (Capella-Gutiérrez et al., 2009) was used for alignment trimming. A phylogenetic tree for each gene was built using the maximum-likelihood approach implemented by PhyML v3.0 (Guindon and Gascuel, 2003). The search for positive selection in codeml is done by comparing the log-likelihood values of codon evolution models that do not allow sites with positive selection (M1a, M7, and M8a) with the values obtained from the more general nested models that also allow for site classes with positive selection occurrence (M2 and M8, respectively). The *p*-values are calculated as 2Δl (twice the difference in the likelihood of the two nested models evaluated) based on the χ^2^ distribution with 2° of freedom for nested models M1a/M2 and M7/M8 and 1° of freedom for nested models M8a/M8. This being a multiple testing scenario, corrected *q*-values from the list of *p*-values obtained for all groups evaluated using the given nested model pair were determined (Hongo et al., 2015).

### Core and Pan-Genome Analysis

To decipher the core genome content of the outlier strains, the get_homologues pipeline was used with OrthoMCL (OMCL) at 75% query coverage as well as sequence similarity (Contreras-Moreira and Vinuesa, 2013). The pan-genome was obtained using OrthoMCL methods and at default parameters (with *t* = 0). The core and pan-genomic contents were determined for all the *Pseudomonas* strains (*n* = 78), classical (*n* = 64), and outliers (*n* = 14) separately. The pangenome for 78 strains was used to find out the genes exclusively present in all the outliers (*n* = 14) that were absent in all the classical strains (*n* = 64). The core and pangenome for all groups were annotated against the COG database by rpsblast (*e*-value 0.001) using WebMGA server (Wu S. et al., 2011).

### Functional Annotations of the Outlier Strains

To determine the functional variability across the 14 outlier genomes in the PA7 clade, the comparative genomic analysis was performed based on proteins and metabolic pathways. The ORFs were predicted using Glimmer-3 and annotated by KAAS (KEGG Automatic Annotation Server) (Moriya et al., 2007) using Bi-directional Best Hit (BBH) algorithm. Further, the metabolic pathways were reconstructed for the outlier genomes using MinPath (Minimal set of Pathways) (Ye and Doak, 2009) to obtain the minimal set of pathways. COG categories were also assigned to the proteome of all the outlier strains by rpsblast (*e*-value 0.001) against the COG database using WebMGA server. The matrix thus generated was plotted as a heatmap with dual dendrogram in R.

### Pathogenic Potential, Resistance Profile and Virulence Factors of Outlier Strains

The genomic data of all the outlier strains were annotated for pathogenic protein families using PathogenFinder and the probability of being a pathogen was calculated using the whole genome data (Cosentino et al., 2013). Resfinder (Kleinheinz et al., 2014) was used to determine the acquired resistance genes in the outliers. Virulence factor database (VFDB) (Chen et al., 2005) was used for deciphering bacterial virulence factors.

### Protein–Protein Interactions

For determining the major key regulatory proteins that are specific to particular clade and can act as potential drug targets in the classical and outlier strains, the interactome of the core genes was analyzed. The protein–protein interaction (PPI) model was constructed using STRING Database v10.5 (Szklarczyk et al., 2017). *Pseudomonas aeruginosa* was selected as the reference organism for PPI construction. The core genes of both the groups were used as input and queried against *P. aeruginosa* PPIs present in the STRING database. The PPI networks were visualized using Cytoscape Version 3.3.0 (Shannon et al., 2003). Network analyzer plugin was used for determining the statistical and functional significance of the PPI networks, calculated using different parameters such as the probability of degree distribution, average clustering coefficient and average neighborhood connectivity as described in previous studies (Albert and Barabasi, 2002; Gupta et al., 2016; Kumar et al., 2017).

### Statistical Analyses of the Network

The degree of probability distribution, *P*(*k*), of a network defined by *P*(*k*) = *nk/N*, which is the ratio of the number of nodes having a *k* degree in the network (*nk*) to the size of the network (*N*), was used to capture the network structure, identification of hubs, and modular organization of the network. The constructed network obeyed the power law, *P*(*k*) ∼*k*^-γ^, indicating the scale-free nature of the network, where γ is an order parameter that identified the different topological structure of a scale-free network. The clustering coefficient *C*(*k*), which is defined by *C (k) = 2E/[k (k - 1)]* is the ratio of the number of edges *E* of the node having a *k* degree with neighbors to the total possible number of such edges, *[k (k - 1)]/2* which is a measure of the topological structure of the network (Watts and Strogatz, 1998). The average clustering coefficient *C*(*k*) identifies overall organization of formation of clusters in the network. Similar to *P*(*k*), *C*(*k*) may depend on network size and characterizes various properties of the network: (i) for scale-free and random networks where *C*(*k*) is independent of *k, C*(*k*) ∼ constant, and (ii) for hierarchical networks where *C*(*k*) follows power-law scaling behavior, *C*(*k*) ∼*k*^β^ with β ∼ 1. The neighborhood connectivity of a node is the number of neighbors connected to it and characterizes the correlation pattern of connectivity of interacting nodes in the network. This connectivity correlation would be measured by defining a conditional probability *P(k*′_n_*|k*_n_) which is the probability of making a link from a node having degree *k*_n_ to another node of degree *k*′*_n_*. Then, the average neighborhood connectivity of nodes with connectivity *kn* is given by *C*_n_ (*k*_n_) = Σ*_k_*_′n_ *k*′_n_*P* (*k*′_n_|*k*_n_) ∼*k*_n_^-α^ (Girvan and Newman, 2002) following a power law scaling behavior with α < 1 for most of the real networks (Maslov and Sneppen, 2002). If *C*_n_(*k*_n_) is an increasing function of *k*_n_ (for negative values of α), then the topology of the network shows assortative mixing (Almaas, 2007) where nodes with a high number of edges per node (high-degree nodes) have an affinity to connect to other high-degree nodes in the network. However, with positive values for α is the signature of the network having a hierarchical structure, where low-degree nodes tend to connect high-degree hubs (Almaas, 2007) and few high-degree hubs present in the network try to control the low-degree nodes.

## Results and Discussion

### Genomic Features Among Outliers

The sequences obtained for the strain CR1 were assembled into two replicons of sizes 6,138,025 bp (Chr 1) and 61,310 bp (pCR1). In order to circularize both the replicons, the ends of the assembled contigs were mapped using BLASTn (Altschul et al., 1990). An overlapping region of 19,971 bp in case of the chromosome and 14,506 bp in the plasmid were detected and removed. The chromosome and plasmid were found to be 6,118,054 and 46,804 bp, respectively, making a total genome size of 6,164,858 bp (6.16 Mbp) (Figure 1A,B). CR1 and PA7 had the complete set of 107 essential marker genes and all the draft genomes were more than 96–98% complete based on essential marker gene approach.

The average genome size of *P. aeruginosa* outliers was 6,539,166 bp ± 233906 bp, with strain AZPAE14941 showing the maximum size (6,881,480 bp) and minimum in strain CR1 (6,164,858 bp) (Supplementary Figure S1). The present results show that the genome size of strain CR1 is smallest among the outlier strains, primarily due to the lack of type I restriction-modification system and mercury resistance cluster (RGP63 and RGP79). In contrast to PA7 (Roy et al., 2010) three RGPs (RGP4, RGP78, and RGP79) having characteristic phage signatures and a putative integrated plasmid (RGP75) were also absent in CR1. Out of the 18 RGP reported exclusive to PA7 (Roy et al., 2010), 13 were present in CR1. As the missing regions mostly coded for resistance to antibiotics and phage-related elements it can be reasonably ascertained that resistance is acquired by the organism under stress in a clinical setup that is missing in the environmental strains. A similar scenario was observed in the genome of an environmental isolate of classical clade- strain M18 (6,327,754 bp) that was closely related to clinical strain LESB58 (6,601,757 bp) but had a smaller genome and was more susceptible to several antimicrobial agents (Wu D. Q. et al., 2011). Based on this, it can be hypothesized that CR1 type genomes are the progenitors of PA7 type genomes and might have acquired multi drug resistance probably in a hospital environment or hospital waste disposal site. Our hypothesis gains support from the fact that an outlier strain CLJ3 that was resistant to antibiotics was isolated after antibiotic administration from the same patient from which CLJ1 was isolated (Elsen et al., 2014). This, strengthens the view that the environment shapes the genomic content of an organism (Kumar et al., 2017). Among all the outliers, only strain CR1 possessed a plasmid of size 46,804 bp with GC content of 59.2% and approximately 45% of ORFs in the plasmid coded for hypothetical proteins. The presence of plasmid within the strain CR1 suggested horizontal gene transfer (HGT) mediated acquisition as none of the outliers reported in the literature possessed any plasmids. Further, the percentage GC content of the plasmid (59.2%) is well below the average GC content (66.5%) of outliers’ genome and members of classical strains of *P. aeruginosa* (Mathee et al., 2008) suggesting its extra-chromosomal origin.

Outlier strains (*n* = 14) possessed a wide variety of CRISPR elements, with a maximum number of CRISPR arrays (*n* = 5) in case of CR1 and AZPAE14901 (Table 1). The presence of a large number of CRISPR elements suggested that these outliers perhaps were vulnerable to bacteriophage attacks that led to the incorporation of CRISPR elements. Eleven outlier strains showed phage protein signatures from *Pseudomonas* phage Pf1 (NC_001331), *Pseudomonas* phage YMC11/02/R656 (NC_028657) and 8 strains showed the presence of phage proteins from *Pseudomonas* phage F116 (NC_006552). The abundance of these phage proteins within the outliers suggested the common phage pool for the *P. aeruginosa* outlier strains. However, a prophage region showing similarity to proteins (Supplementary Table S3) of various phages that are known to infect bacteria associated with soil or environmental samples were found only in CR1 strain indicative of its environmental origin.

**Table 1:** General genomic attributes of 14 outlier strains of *P. aeruginosa*: ANIm values were calculated between CR1 and other outliers.

Strain	Origin (Country)	Genome size	Number of contigs	Genes	Sequence type	tRNAs	rRNAs (5S, 16S, 23S)	GC content (%)	CRISPR arrays	Prophage regions	Completeness (Genes/%)	ANIm (%)	DDH^∗^(%)	Reference
PA7	Burn wound (Argentina)	6588339	1	6302	1195	75	4,4,4	66.5	3	4	107 (100)	98.97	91.1	Roy et al., 2010
CR1	Rhizosphere (India)	6164858	2	5838	3198	76	4,4,4	66.8	5	2	107 (100)	100	100	This study
ATCC 9027	Otitis (Australia)	6362326	80	6121	2230	69	1,1,1	66.6	1	2	106 (99.06)	99.33	93.9	Mai-Prochnow et al., 2015
AZPAE14901	Abcess/Pus (India)	6881448	232	6709	2212	63	1,1,1	66.2	5	2	105 (98.13)	99.32	86.6	Kos et al., 2015
AZPAE14941	Peritoneal (Hong Kong)	6881480	183	6722	Not Assigned	66	2,1,1	66.0	3	2	105 (98.13)	98.94	85.7	Kos et al., 2015
AZPAE15042	Urinary (Germany)	6636169	143	6470	2211	69	1,1,1	66.3	3	1	105 (98.13)	99.36	90.7	Kos et al., 2015
EML528	Unknown (Germany)	6365411	53	6064	3043	70	2,1,1	66.6	4	1	107 (100)	98.95	91.7	Boukerb et al., 2015
EML545	Unknown (Germany)	6442089	64	6142	2228	68	3,3,3	66.6	3	2	107 (100)	99.37	94.0	Boukerb et al., 2015
EML548	Unknown (Germany)	6333473	120	6088	2230	73	4,4,4	66.6	1	2	103 (96.26)	99.34	93.8	Boukerb et al., 2015
WH-SGI-V-07055	Clinical (United States)	6757340	102	6532	2023	67	7,1,1	66.4	3	5	107 (100)	98.90	89.6	van Belkum et al., 2015
WH-SGI-V-07064	Clinical (United States)	6336003	87	6066	1195	68	6,1,1	66.7	2	3	105 (98.13)	98.96	93.8	van Belkum et al., 2015
WH-SGI-V-07072	Clinical (United States)	6373176	88	6103	1195	68	6,1,1	66.6	2	1	105 (98.13)	98.95	93.5	van Belkum et al., 2015
WH-SGI-V-07370	Clinical (France)	6581092	174	6358	2047	67	6,1,1	66.4	2	2	105 (98.13)	98.94	91.1	van Belkum et al., 2015
WH-SGI-V-07618	Clinical (United States)	6845114	159	6622	2023	68	9,1,1	66.2	3	4	105 (98.13)	98.90	88.7	van Belkum et al., 2015

*^∗^GGDC server was used to calculate genome to genome distance among outliers.*

For differentiating these outliers based on their genetic background and deciphering the sequence type, a combination of DNA sequences of housekeeping alleles *acs-5, aro-8, gua-3, mut-5, nuo-1, pps-11*, and *trp-3*, and their whole genome data was used^2^. Amongst the outliers (*n* = 14), 11 were assigned known sequence type and it was found that strain CR1, AZPAE14941, and EML528 corresponds to an unknown sequence type (Table 1), highlighting a possibility of novel sequence type for these three outlier strains. Subsequently, strain CR1 and EML528 were assigned new sequence types, while no sequence type was assigned to AZPAE14941 due to its allele profile being incomplete (Table 1).

### Conjugative Plasmid pCR1

The genes encoded by pCR1 had no significant sequence similarity with the genomic sequence of the outliers making the genes encoded by pCR1 specific to strain CR1. Although it had 45% hypothetical proteins, pCR1 had all proteins required for replication (B7D75_29005), plasmid maintenance (ParA: B7D75_28995, ParB: B7D75_29000 KikA protein: B7D75_29260) and conjugation (TraL: B7D75_29275, T4SS B7D75_29180-29235) (Figure 1B) that made up the plasmid backbone. Additionally, a HigB/A toxin/antitoxin (TA) (B7D75_29125/B7D75_29130) system that is shown to influence the virulence factors pyochelin, pyocyanin, and biofilm formation in *P. aeruginosa* (Wood and Wood, 2016) was also detected. pCR1 showed an overall identity (Iden) of 97% with a query coverage (QC) of 76% with a newly sequenced plasmid p14057 having KPC resistance (BLASTn; NCBI NR database). It also contained VirB/D4 type IV secretion system (T4SS) gene cluster (B7D75_29180-29240), showing similarity with the T4SS present on the chromosome of *P. savastanoi* (QC: 83% and Iden.: 100%; Accession: FN645745.1) and to T4SS on the plasmid of *P. syringae* B76 (QC: 65% and Iden: 68%; Accession: JQ418525.1). VirB/D4 type T4SS mediates the transfer of DNA-protein complexes and other proteins like virulence factors to the host cell by conjugation (Fronzes et al., 2009; Wallden et al., 2010). The putative relaxase (B7D75_28990) on pCR1 in association with the T4SS system might be responsible for rolling circle replication-mediated conjugation (Chandler et al., 2013) in strain CR1. Notably, this plasmid had no identity to the conjugative integrated pKLC102 region (RGP75) of the strain PA7. Additionally, a conjugal transfer protein (B7D75_29255) having *yadA* like anchor protein domain (LPXTG motif) similar to the putative adhesin on the plasmid of strain PA3448 isolated from blood of human (QC: 100%, Iden.: 74%; Accession No: OGX61696.1) was found in pCR1. This protein belongs to the class of trimeric autotransporters adhesins (TAAs) and is characterized as a virulence factor by BLASTp based on similarity with VFDB protein dataset, increasing the capability of adhesion to both biotic and abiotic surfaces and auto-aggregation leading to biofilm formation (Linke et al., 2006). The genes for antibiotic resistance often reported from clinical *P. aeruginosa* plasmids were absent.

### Consensus Phylogenomic Clustering of Outliers

**FIGURE 2:**
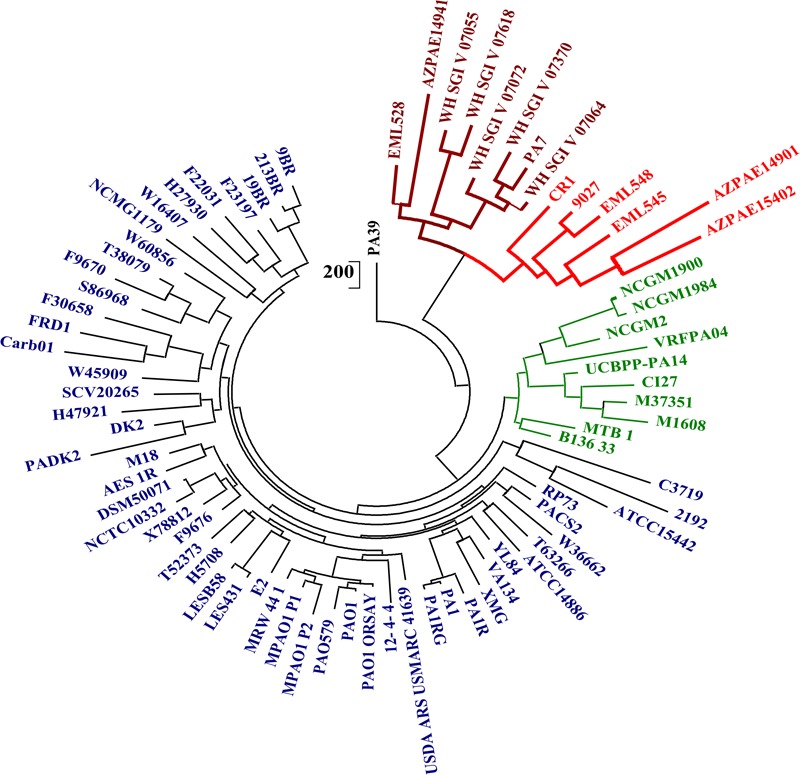
Phylogenetic analysis of *P. aeruginosa* strains using pan-genome matrix. The consensus pan-genome matrix was generated using the intersection of pan genes predicted by COG and OMCL algorithms to construct a parsimony tree using PARS from the PHYLIP. Three major clusters represented by PAO1 (Blue), UCBPP-PA14 (Green), and PA7 (Maroon) were observed. The outlier clade represented by PA7 was split into 2 clades one with a representative genome of PA7 (Maroon) and second by strain CR1 (red).

To demarcate the outlier strains of *P. aeruginosa*, the phylogenetic and phylogenomic analyses were performed by using 400 conserved marker genes, ANIm, core genome alignment, and pan-genome gene presence/absence matrix. All the analyses revealed three clades with representative genomes of PAO1, UCBPP-PA14, and PA7. It corresponded with the phylogeny obtained by Freschi et al., 2015. CR1 was the member of the PA7 clade with members that are completely devoid of type III secretion system. Strains in the outlier clade shared nearly 94% identity with the classical strains whereas, within the clade, the percentage identity increased up to 98–99%, suggesting the higher similarity in outliers’ genomic repertoire. A similar trend of clustering was observed in the phylogeny based on 400 ubiquitous conserved bacterial genes, pan-genome matrix using maximum likelihood method (Guindon and Gascuel, 2003) (Figure 2 and Supplementary Figures S2, S3) and unrooted tree based on core genome alignment (Supplementary Figure S4). This confirmed the high level of genomic relatedness among the classical strains and outliers yet reflecting the two groups to be different. The results from our analyses suggest that the whole genome-based phylogeny methods should be used for delineating the closely related strains which are found to be 100% similar on the basis of 16S rRNA gene sequence.

The dendrograms based on ANI, tree based on core-genome alignment and pangenome matrix provided better resolution of phylogenetic distance among the clades as these strains differed on the basis of the pan-genomic content and SNPs in the core genome alignment and in case of 400 conserved genes, the resolution was less due to high sequence identity among the 400 marker genes (98–99%). Based on this the outlier clade was distinctly represented by two sub-clades one with the representative complete genome of CR1 with draft genomes of strains ATCC9027, EML548, EML545, AZPAE14901, and AZPAE15042 and the second clade had PA7 as the representative with the genomes of EML528, AZPAE14941, WH-SGI-V-07055, WH-SGI-V-07618, WH-SGI-V-07072, WH-SGI-V-07370, and WH-SGI-V-07064. Based on the genetic divergence, we propose two sub-clades within the outliers.

### Regions of Genomic Plasticity (RGP) in Strain CR1

Previous studies have shown the presence of various regions of genomic plasticity (RGP) within *P. aeruginosa* strains (Mathee et al., 2008; Roy et al., 2010; Jani et al., 2016). As the genome of strain CR1 is nearly 99% similar to PA7, there were large regions of DNA that were 100% similar (Supplementary Figure S5). At the time of analysis 87 RGPs (numbered 1 to 97) had been reported in *P. aeruginosa* strains (Klockgether et al., 2011; Boukerb et al., 2016). Out of the 87 RGPs, 32 were missing in strain CR1 and it harbored eight new RGPs which were added to already known 87 RGPs (numbered RGP98-RGP105; Supplementary Table S4). Although the sequence similarity of strain CR1 and PA7 based on ANIm was 98.97%, strain CR1 harbored 11 prominent regions (eight new RGPs and differences in three already known RGPs) having features not reported earlier. Two previously known regions (RGP5 and RGP72) had particular ORFs in strain CR1 which were absent in strain PAO1, UCBPP-PA14, and PA7. The third region, RGP58 was found to be missing in PA7 but was present in CR1. The entire list of RGPs has been compiled (Supplementary Table S4) and the strain-specific features of CR1 have been listed (Table 2 and Supplementary Figure S6). Replacements islands were identified in RGP9, RGP31, RGP60, and RGP68 that had the strain-specific genes for flagellin glycosylation, *O*-antigen gene cluster, *pilA* gene, and pyoverdine synthesis gene cluster, respectively. RGP63 and RGP64 had homologous ORF disrupted by insertion in strain CR1. The ORFs included in the RGPs were identified and numbered as per the NCBI locus tag for strain CR1 (B7D75) and PA7 (PSPA7). The flanking loci (conserved) of each RGP called anchors in strains PAO1 and UCBPP-PA14 were also identified as listed in Supplementary Table S4.

**Table 2:** Details to RGPs found in strain CR1: number of ORFs, GC content, tRNA, integrase and transposase genes, major annotation, similarity/origin, and putative functions.

GI (RGP)	Size (bp)	%G+C	tRNA	Integrase	Transposase	Major annotations	Similarity	Putative functions
RGP98	4,694	55.86	No	No	Yes	Mobile Genetic Elements, Helicase, Bacterial Partitioning Protein	Helicase and Partitioning proteins have origins from *Bordetella* and *Acidhalobacter*	Transposition of genetic elements
RGP99	41,377	64.21	Yes	Yes	No	Phage-related proteins, Hypothetical proteins	Major hits from *Stenotrophomonas* Phage S1	Recombination (presence of attL and attR sites)
RGP100	7,638	64.68	No	No	No	Type I secretion protein (TolC), ABC Transporters, ATP-binding proteins, haemolysin D, YceK/YidQ family proteins	Non-*Pseudomonas aeruginosa* origin	TISS (pathogenic potential)
RGP101	7,313	51.21	No	Yes	No	Hypothetical proteins	Classical strains of *P. aeruginosa*	Hypothetical proteins
RGP102	11,296	66.10	No	No	No	Hypothetical proteins	Non-*Pseudomonas* origin; *Burkholderia* and *Xylella*	Unknown
RGP103	14,440	65.93	Yes	Yes	No	Formaldehyde activating enzyme, aldehyde dehydrogenase; aldo/keto reductase, Gluconate dehydratase.	*Pseudomonas citronellolis*	Aldehyde Detoxification; D-gluconate and ketogluconate metabolism
RGP104	12,652	58.79	Yes	No	No	Amino acid permease, arginine/agmatine antiporter, SAM-dependent methyltransferase	Origin from classical strains of *P. aeruginosa*	Acid tolerance/resistance
RGP105	4,251	60.50	No	No	No	*rhiA, rhiB* genes, transcriptional regulators	Origin from soil inhabiting bacteria like *Rhizobium*	Rhizosphere expressed genes

These regions of genomic plasticity as observed in strain CR1 might provide various strain specific advantages to the organism. For instance, RGP72 in strain CR1 harbored genes for galactonate metabolism from the non-*Pseudomonas* origin. Genes involved in D-gluconate, ketogluconate metabolism and choline degradation that were absent in PA7 were present in RGP103. The ORFs in this region had 87–93% similarity with *P. citronellis*, indicative of HGT. Furthermore, the RGP104 region had an arginine/agmatine antiporter that has a major role in maintaining the internal pH of the cell and also found in enteric pathogenic bacteria (Fang et al., 2010). All these regions are important in maintaining the osmotic balance of cell, protect it against reactive oxygen species (ROS) and provide continuous energy to the cell by supplying intermediates for the Entner-Doudoroff (ED) pathway and prevent starvation (Buch et al., 2008; Ghasempur et al., 2014). RGP100 contained genes for type I secretion system (T1SS) and RGP58 coded for type VI secretion system proteins that can impart a pathogenic potential to strain CR1 (Hood et al., 2010; Thomas et al., 2014). BLASTn analysis revealed that ORFs in this region had a non-*Pseudomonas* origin. ORFs in replacement island RGP31 had genes for *O*-antigen biosynthesis protein that is known to be the most variable component of the LPS (Steimle et al., 2016). It showed only 66% identity (QC: 55%; *e*-value: 1*e*-105) to its homolog present in PA7 and was more closely related to strain NCMG1179 (QC: 99%; identity: 98%). Regions like RGP99, RGP98, and RGP101 carrying transposases, partitioning proteins, and phage proteins could help in recombination and efficient transposition of the genetic elements across the genus. RGP105 had genes coding for rhizosphere expressed (*rhi*) gene operon which has been linked with plant-microbe interactions in *Rhizobium leguminosarum* (Giordano, 2015), thus supporting the environmental origin in CR1 strain. Rhizosphere expressed genes were absent in all the clinical genomes.

### Genome-Scale Positive Selection Detection

A total of 5457 orthologous groups were analyzed for gene undergoing positive selection, after filtering 128 genes that were identified to exhibit significant evidence for homologous recombination (FDR < 10%). Among these, 20 genes were found to be subjected to strong selection pressure (Table 3) with ω > 1 (ω equal to the ratio of dN to dS for amino acid sites under positive selection). Genes coding for proteases, bacterial transporters, DNA replication-stabilization, and hypothetical proteins with unknown function in the *P. aeruginosa* outlier genomes were under positive selection (Table 3). A carbohydrate binding protein with metzincin protease and transglutaminase cysteine protease may have a role in biofilm formation and dispersal (Passmore et al., 2015; Rybtke et al., 2015). Epoxide hydrolase that has been shown to decrease mucociliary transport and restrict bacterial clearance from the lung (Hvorecny et al., 2018) was observed as undergoing positive Darwinian selection. In addition to these, Metallo-β-lactamase that can degrade β-lactam antibiotics (Palzkill, 2013) and peptidase M48 with dual nature of chaperone and metalloprotease that are believed to mediate tissue penetration and infection (Maredia et al., 2012) were also positively selected. Further, *oprD* porin was also found to be under positive selection, and it has been shown to carry mutations in classical strains of *P. aeruginosa* and has been regarded as a mosaic gene (Lynch et al., 1987; Pirnay et al., 2002) that plays a role in antibiotic resistance against carbapenems due to its reduced uptake (Li et al., 2012). Various transporters like lysine exporter, sulfate transporter, amino acid permease, and transporter for branched-chain amino acids were also positively selected (Table 3). They provide a continuous supply of amino acids to the cell and prevent starvation (Tralau et al., 2007). Although these transporters are important for maintaining adequate nutrient levels, the branched-chain amino acids (BCAA) are an important link between the metabolic state of the cell and virulence in *Staphylococcus aureus* by acting as co-repressors of global transcriptional regulators (Kaiser et al., 2015). These proteases and transporters have a dual role in survival and pathogenicity of these outliers and can play a key role in shaping the pathogenic potential of these strains in the course of evolution.

**Table 3:** The list of genes under positive natural selection [ω represents the value of dN/dS, *p*-value: probability value; *q*-value: false discovery rate (FDR < 10%)].

Gene	Function	Cluster name	ω	*p*-value	*q*-value
**Proteases and hydrolases**					
Carbohydrate-binding protein with metzincin domain (B7D75_11240)	Protease secreted by biofilm of *P. aeruginosa*	ORTHOMCL2836	29.07471	0	0.000844
Epoxide hydrolase (B7D75_07235)	Virulence factor; hydrolysis of CFTR in lungs	ORTHOMCL5445	30.65061	0	0.000576
Transglutaminase cysteine protease (B7D75_11075)	Role in biofilm formation and dispersal	ORTHOMCL2866	23.91616	0.000006	0.004772
Metallo β-lactamase fold metallo-hydrolase (B7D75_21135)	Degrade beta-lactam antibiotics	ORTHOMCL4367	21.31702	0.000023	0.010209
Outer membrane porin, OprD family (B7D75_11385)	Mutation in *opr*D mechanism for carbapenem resistance	ORTHOMCL2807	18.80725	0.000082	0.028645
Peptidase M48 (B7D75_26190)	Chaperone and metalloprotease	ORTHOMCL1727	17.31162	0.000174	0.045382
**Bacterial transporters**					
LysE family translocator (B7D75_10555)	Lysine exporter	ORTHOMCL2297	21.92155	0.000017	0.00845
Hydrogen peroxide inducible genes activator (LysR family) (B7D75_26850)	Expression of hydrogen peroxide-inducible genes	ORTHOMCL481	25.94273	0.000002	0.002425
High affinity sulfate family inorganic anion transporter (B7D75_00150)	Sulfate transporter	ORTHOMCL1053	22.29005	0.000014	0.00845
Branched-chain amino acid transport system II carrier protein (B7D75_16930)	Transport system for branched-chain amino acids (BCAAs; Ile, Leu, and Val)	ORTHOMCL3746	17.34394	0.000171	0.045382
Amino acid permease (B7D75_21325)	Amino acid transporters	ORTHOMCL4632	17.92312	0.000128	0.039327
Type IV pilus secretin PilQ (B7D75_26155)	Involved in type IV pilus formation, competence for transformation, type III secretion, and type II secretion	ORTHOMCL1798	20.61727	0.000033	0.013371
ProQ activator of osmoprotectant transporter ProP (B7D75_12075)	Transport betaine and other osmoprotectants	ORTHOMCL2663	18.45609	0.000098	0.032009
**DNA stabilization and replication**					
Nuclease SbcCD subunit C (B7D75_21985)	Double stranded exo- and endonuclease	ORTHOMCL4686	24.01605	0.000006	0.004772
ATP-dependent DNA helicase (B7D75_08610)	DNA replication	ORTHOMCL2136	21.94135	0.000017	0.00845
Error-prone DNA polymerase (B7D75_21945)	DNA replication	ORTHOMCL4522	17.55637	0.000154	0.044617
**Hypothetical protein**					
hmga2e, putative (B7D75_11510)	Unknown	ORTHOMCL2780	21.86925	0.000018	0.00845
Hypothetical protein (B7D75_05465)	Unknown	ORTHOMCL1549	38.14207	0	0.000027
Hypothetical protein (B7D75_14605)	Unknown	ORTHOMCL3251	27.56469	0.000001	0.001347
Terminase (B7D75_05835)	Viral terminase	ORTHOMCL6048	20.30993	0.000039	0.014478

### Core-Pan-Genome Dynamics and Functional Analysis

**FIGURE 3:**
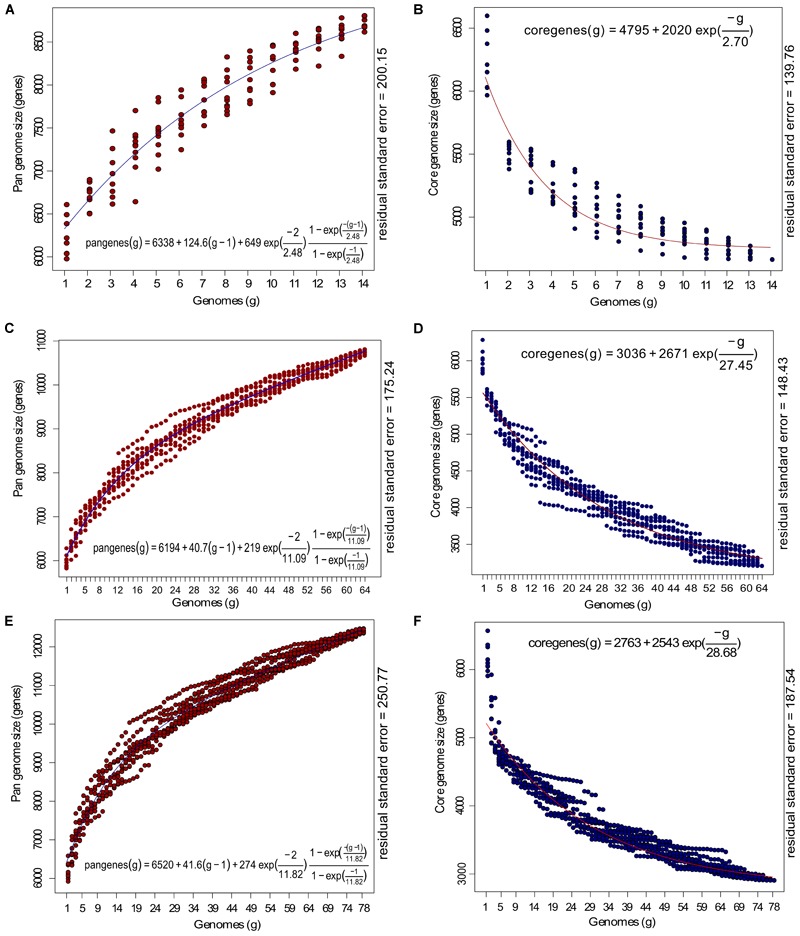
Estimation of core and pan-genome of *P. aeruginosa* strains with Tettelin fit (blue: pangenome; red: core-genome). **(A,B)** Represents the estimate of the pan and core genome of 14 outlier strains; **(C,D)** represents the estimate of core and pan-genome of 64 *P. aeruginosa* strains included in the current study and **(E,F)** represents estimate of the core and pan-genome curves for 78 genomes. The *x*-axis represents the number of genomes (g) while the *y*-axis represents the core genome and pan-genome size (number of genes). The equation for estimating pan and core-genome size according to Tettelin fit are given with the respected graphs.

To examine the core-pan dynamics, we made three groups: outliers (*n* = 14), classical strains (*n* = 64), and all strains (*n* = 78). The groups were analyzed for their core genome content using OrthoMCL with 75% query coverage and sequence similarity and pangenome at default parameters in get_homologues and plotted with Tettelin fit (Figure 3) (Tettelin et al., 2005). The analysis revealed a core genome size of 4,708 genes and 10,429 non-redundant pangenes within 14 outlier genomes. This was further followed by the analysis for core and pan-genome of classical strains (*n* = 64). The core and pan-genome size in case of 64 classical strains were 3199 and 16705 genes, respectively, and as soon as 14 outliers were added, the core genome shrank to 2885 genes while the pangenome increased to 19736 genes, clearly signifying the expansion of pangenome. The frequency of core and accessory genes between outliers and classical strains were compared using the Chi-square test of independence, and were found to be significant [χ^2^(1) = 51.63, *p* < 0.001]. Further, the outlier genomes were more similar than the classical strains as reflected by the high prevalence of core genes in them (44.60%) *vis-a-vis* classical strains (19.15%). The core genome size in outliers was estimated using Tettelin fit under the assumption of 64 genomes (*g* = 64) for outlier group. This suggested that the core genome in case of hypothetical 64 outlier genomes (using the equation in Figure 3B) would be ∼4700 genes. This supports the observation that all the 14 genomes are highly identical to each other and the majority of the genes are conserved as core genes and adding more genomes will not decrease the core genome size drastically. On the contrary, there was a decline (9.82%) in the core genes and increase (15.35%) in the pangenes of the classical strains when all the genomes (*n* = 78) were subjected to analysis supporting the fact that these two groups are significantly different.

**FIGURE 4:**
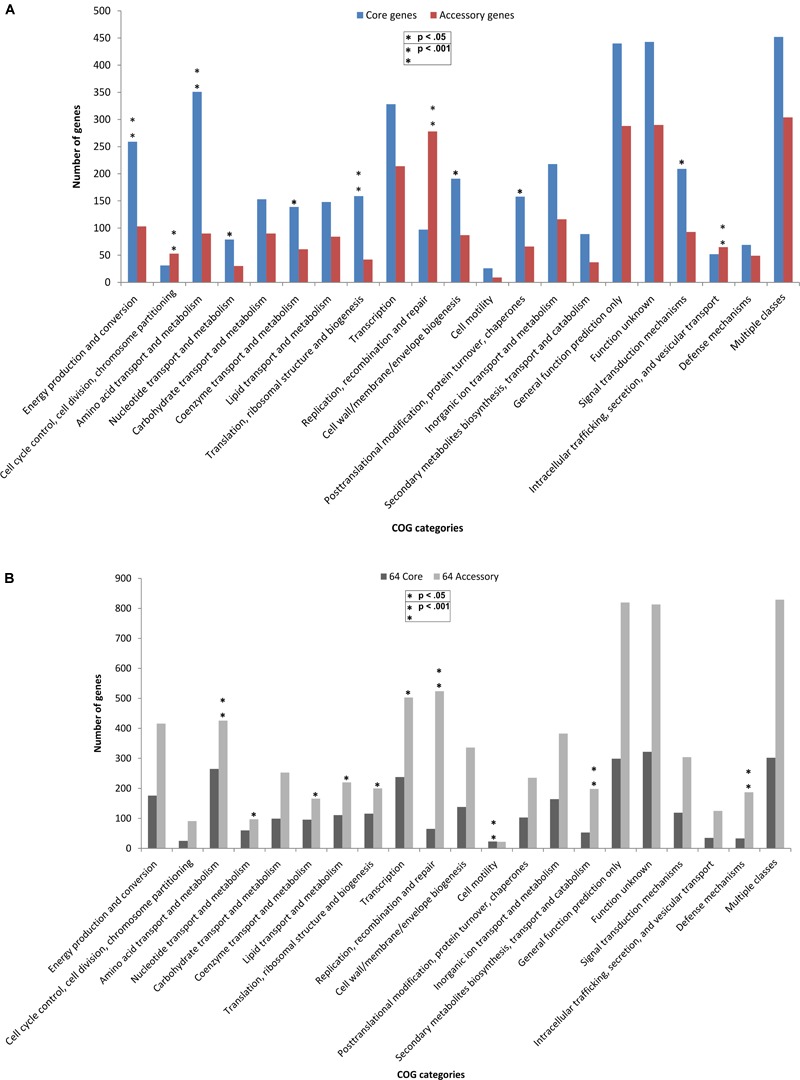
Comparative functional analysis based on COG categories between core and accessory genes in **(A)** outlier strains (*n* = 14) and **(B)** classical strains (*n* = 64). COG-based binning of core genes and accessory genes. The abscissa denotes different COG functional categories. The ordinate denotes the number of genes in each COG category. Four COG functional categories (RNA processing and modification, Chromatin structure and dynamics, Extracellular structures, and Cytoskeleton) including only one or without homologs in the COG collection are not displayed. Significant enrichment of gene occurrence in the individual category is marked by asterisks (^∗^*p* < 0.05, ^∗∗^*p* < 0.001; FDR < 10% Chi-square test).

The core and accessory genes in both the groups were annotated for COG (Clusters of Orthologous Groups of proteins) functional categories. The distribution of COG functional categories was remarkably different between core genes and accessory genes. In comparison to the core genome in 14 outliers, the accessory genes encoding proteins involved in replication, recombination and repair, cell cycle control, cell division, chromosome partitioning and intracellular trafficking (p-value < 0.001 and FDR < 0.03) were more in number as compared to the core genes (Figure 4A). Replication, recombination and repair, and cell motility categories had more genes in accessory and core categories, respectively, in both the outlier and classical groups. This can be attributed to the fact that these outlier and classical strains are from diverse niches and therefore possessed a large number of accessory genes for maintaining replication and cell division in various environments, at the same time, maintained a core set of genes required for motility. We also found three genes of the replication gene category to be under positive selection which can be a major cause of generation of advantageous variants for the population and make these organisms ubiquitous. However, the COG categories responsible for functions like amino acid metabolism, translation, ribosomal structure and biogenesis, energy production and conversion (p-value < 0.001 FDR < 0.03), post-translational modification, protein turnover, signal transduction mechanisms, nucleotide transport and metabolism, and cell wall biogenesis were significantly (p-value < 0.05 FDR < 0.05) more enriched in the core genes of the outliers while a higher number of accessory genes were found in almost all the COG categories of the classical strains (Figure 4B). The high abundance of gene categories in the core of the outlier genome (n = 14) reflected that they are metabolically similar amongst themselves but distinct from the classical strains.

Although 6276 more pangenes were present in classical strains, comparison of the pangenes in 64 classical and 14 outlier strains revealed 136 genes that were exclusively present in the outliers. Most of these genes were hypothetical. COG annotation revealed 78 genes which belonged mainly to transcription, energy production and conversion, amino acid transport and metabolism and secondary metabolites biosynthesis, transport and catabolism. These in our opinion contributed to the outlier specific genes and hence their presence could be an important parameter to characterize the outliers’ clade. All the outliers had the D-Ara*f* biosynthetic pathway, but it was also present in Exl-A positive strain CF_PA39 (Huber et al., 2016). From the 136 genes we found the complete cluster for enzymes involved in L-Ectoine degradation *viz. doeA* (B7D75_20030; ectoine hydrolase; EC 3.5.4.44), *doeB* (B7D75_20035; N2-acetyl-L-2,4-diaminobutanoate deacetylase; EC 3.5.1.125), and *doeC* (B7D75_09470; aspartate-semialdehyde dehydrogenase; EC 1.2.1.M5). Many environmental isolates use ectoine as a growth substrate (Schwibbert et al., 2011) that also serves as key osmoprotectant (Yu et al., 2017). In addition to this, we found a region specific to all the outliers with genes L-Proline/Glycine betaine transporter (ProP) (B7D75_13890), nitrilase (B7D75_13895), cytochrome c (B7D75_13900) and cytochrome d1 nitrite reductase (B7D75_13905) that help bacteria to cope with osmotic stress in environment (Chen and Beattie, 2008), catalyzes the hydrolysis of nitriles to carboxylic acid and ammonia without formation of free amides, and have roles in hormone synthesis, nutrient assimilation and detoxification of exogenous and endogenous nitriles (Howden and Preston, 2009; Raczynska et al., 2011). All these characteristics were specific to outliers and were absent in the classical strains of *P. aeruginosa*. In addition to being the group-specific genes for the outliers, the presence of these genes also pointed out that the outliers as a group are very distinct in their functional attributes and possess many genes which confer an advantage in surviving under environmental stress.

**FIGURE 5:**
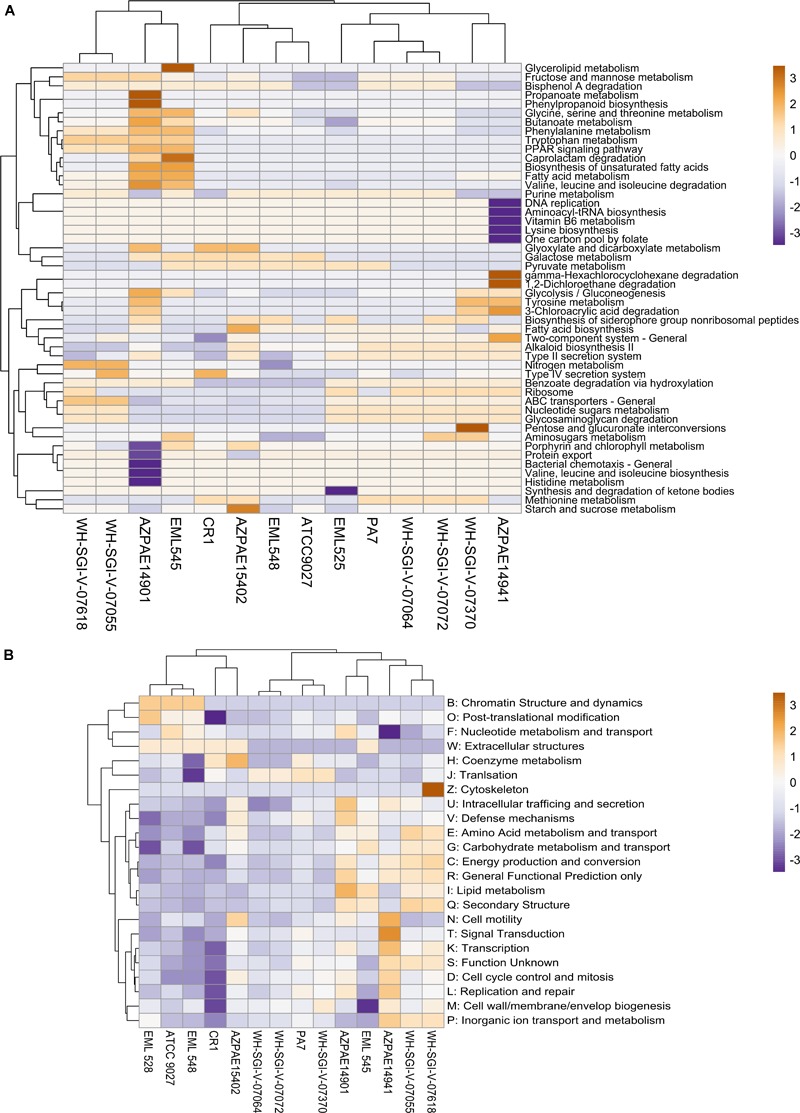
Functional analysis of *Pseudomonas* outlier strains. **(A)** The amino acid sequences of all the 14 genomes were assigned KO number by KAAS server. The protein families were then mapped for a minimal set of pathways using a parsimony approach. The heat map was constructed based on most abundant top 50 subsystems using Pearson correlation. **(B)** The ORF’s were mapped against the COG database using rpsblast. For both the heatmaps rows are centered; unit variance scaling is applied to rows. Both rows and columns are clustered using correlation distance and average linkage.

For deciphering the functional disparities in outliers, the genomes (*n* = 14) were annotated using KEGG orthology and subjected to pathway reconstruction by MinPath. The functional analysis revealed the differential abundance of genes encoding Type IV secretion system, nucleotide sugars metabolism, ABC transporters and pathways linked to energy production and metabolism based on the minimal set of pathways (Figure 5A). This observational difference suggests different metabolic preferences of *P. aeruginosa* outlier strains. Further, T3SS was completely missing- suggesting the genotypic validation for outliers as they were reported not to harbor T3SS (Roy et al., 2010). Interestingly, the analysis based on minimal pathways clearly indicated that CR1 was in the clade of PA7; thus suggesting that common complete metabolic pathways exist in both the strains. However, the clustering in case of COG categories generated a topology similar to the phylogenetic analysis where the two genomes were present in different sub-clades (Figure 5B).

### Virulence Genotype

All the outlier strains (*n* = 14) were found to be highly identical (98.5–99.5%). CR1 strain possessed all the genes required for flagella formation, type IV pili biosynthesis, type IV pili twitching motility, alginate biosynthesis and regulation but lacked a *toxA* gene similar to PA7. It also harbored the *exlA* (B7D75_20920) and *exlB* (B7D75_20915) required for the formation of exolysin A. Apart from the similarity in the genetic repertoire of most of the flagellar assembly genes, strain CR1 possessed *flaB* gene (B7D75_19585) instead of *flaC* gene (PSPA7_4279). These two genes have higher similarity at their N-terminus and comparatively lesser similarity at the C-terminus with an overall query coverage and percentage similarity of 85 and 63%, respectively. Three more outlier strains *viz.*, EML545, WH-SGI-V-07055, and WH-SGI-V-07618 had the same genotype and rest of the outliers had *flaC* gene as in strain PA7 indicating this locus to be distinct in comparison to other genes required for flagella biosynthesis. Recently *flaC* locus from *Campylobacterales* was reported to have a high intrinsic property to activate the innate immune response (Faber et al., 2016). Genes coding for LPS-O antigen in CR1 lacks three genes UDP-*N*-acetylglucosamine 2-epimerase (EC 5.1.3.14; PSPA7_1971), UDP-glucose 4-epimerase (EC 5.1.3.2; PSPA7_1984), and Undecaprenyl-phosphate N-acetylglucosaminyl 1-phosphate transferase (EC 2.7.8-; PSPA7_1985) that can lead to strain-specific lipopolysaccharide in the cell wall of CR1 strain.

Phenazines are key virulence factors that are characteristic of *P. aeruginosa*. Outliers (*n* = 14) had fused *phzA1* with *phzB1* and this can be used as a marker gene to distinguish an outlier strain from the classical strains. Our analysis to identify outlier specific genes from the core-genes which were present in all the 14 outliers and absent from 64 classical strains also confirmed that all the 14 outliers had the fused *phzA1* and *phzB1* (B7D75_03695). Strain CR1 and PA7 were found to harbor complete operons *phzB1-phzG1* (B7D75_03695-03670); *phzA2-phzG2* (B7D75_15445-15415), *phzM* (B7D75_03705), and *phzS* gene (B7D75_03660) but lacked *phzH*. The fused gene does not alter phenazine production, and strain CR1 produced the characteristic blue color pigment in the culture broth which is indicative of the production of pyocyanin. Further, all the outliers barring EML528 had the complete operon *pvsA-pvsE* (B7D75_14020- B7D75_14040) and vibrioferrin receptor *pvuA* (B7D75_14015) that are required for the production of vibrioferrin, a siderophore; which was also reported in agriculturally important *Azotobacter vinelandii* and was previously reported only in marine bacteria (Baars et al., 2016). This may in part explain why the CR1 strain has been known to have the ability to promote the growth of plants (data not presented here). The entire cluster of vibrioferrin was absent in classical strains like PAO1 and UCBPP-PA14. Recently vibrioferrin has been reported in *P. fragi* (Stanborough et al., 2017) but so far vibrioferrin production has not been reported in *P. aeruginosa*. This indicates that all the outliers possess extra siderophore producing genes that are absent in classical strains. This suggests the acquisition of this gene cluster by horizontal gene transfer from other strains of *Pseudomonas* spp. and other bacteria in soil. The genes for pyoverdine were also present in all strains. PA7 is known to produce type II pyoverdine and has the characteristic 966-bp coding region that contained an esterase/lipase protein domain (PSPA7_2860) in strain CR1 (B7D75_13235) (Smith et al., 2005). However, the ferripyoverdine receptor (*fpvA*) was of the type IIb in all strains. Interestingly strain CR1 lacked both hemolytic phospholipase C (*plcH*) and phospholipase D (*pldA*). Rest all factors for quorum sensing systems, regulation by *GacS/GacA* two-component systems and hydrogen cyanide production showed complete synteny to all the complete genomes of *P. aeruginosa*.

**FIGURE 6:**
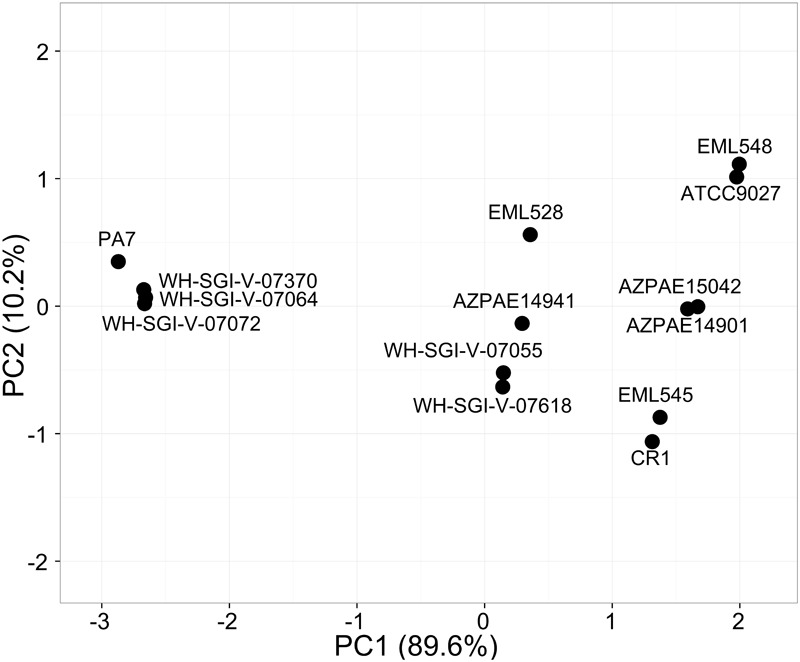
PCA analysis on pathogenic potential of *P. aeruginosa* outlier strains. The PCA- analysis was performed on the four parameters namely number of pathogenic and non-pathogenic families annotated, probability of being a pathogen and input proteome percentage among the 14 strains. PA7 along with WH-SGI-V-07370, WH-SGI-V-07064, and WH-SGI-V-07072 clustered in a different quadrant from the other strains showing high pathogenicity.

The probability of being a pathogen depicting the pathogenic potential of the outliers was in the range of 62–68% among the 14 strains, lowest being for ATCC9027 and EML548 and highest for PA7, WH-SGI-V-07072, and WH-SGI-V-07370. The pathogenicity potential of strain CR1 (66.0%) was significantly higher [χ^2^(1) = 10.24, *p* < 0.005]. The number of pathogenic families annotated in the genome was highest in strain PA7 with input proteome coverage 15.72% and 757 pathogenic families and lowest in case of strain ATCC9027 (Table 4). The principal component analysis that is used to visualize genetic distance and relatedness were performed using four parameters (probability of being a pathogen, input proteome coverage, matched pathogenic family and matched non-pathogenic families) among the 14 genomes. The PCA bi-plot (Figure 6) clearly showed that strain PA7, WH-SGI-V-07370, WH-SGI-V-07064, and WH-SGI-V-07072 formed a distinct compact cluster, separate from other strains indicating them to be more pathogenic than other outlier strains. Interestingly even among the outliers, CR1 clustered in the group of less virulent strains represented by strain ATCC9027 possibly due to its non-clinical origin and less pathogenic proteins. Although pathogenicity is context dependent and strains of PA7 clade have been shown to be avirulent in mammalian infection models but were pathogenic in invertebrate and plant infection models (Hilker et al., 2015). Insights into the pathogenic protein families clearly indicated that these strains were less pathogenic when compared to the strains of classical clade having a higher probability of being a pathogen in the range of 70.8–76.4% (Supplementary Table S5) but have a significant number of pathogenic families in their genomes.

**Table 4:** Pathogenic potential of the 14 outlier strains.

Strain	Probability	Input proteome coverage (%)	Matched pathogenic families	Matched not pathogenic families
ATCC9027	0.617	5.61	238	92
EML548	0.615	5.68	239	93
CR1	0.660	5.51	244	69
AZPAE14901	0.638	5.15	248	82
EML545	0.656	5.42	249	71
AZPAE15042	0.639	5.45	252	83
EML528	0.640	8.75	383	125
AZPAE14941	0.653	7.84	387	115
WH-SGI-V-07618	0.663	7.92	395	107
WH-SGI-V-07055	0.661	8.04	395	109
WH-SGI-V-07072	0.675	15.35	719	179
WH-SGI-V-07064	0.674	15.51	721	180
WH-SGI-V-07370	0.676	14.92	730	180
PA7	0.672	15.72	757	191

*Principal component analysis was performed on the four factors viz. The probability of being a pathogen, number of pathogenic families, input proteome coverage for pathogenic family and matched non-pathogenic families.*

### Antibiotic Resistance of Outlier Strains

Resfinder-v2.1 predicted the presence of acquired resistance genes for β-lactam resistance, aminoglycoside resistance, phenicol resistance, and sulphonamide resistance. Interestingly, all the strains (*n* = 14) including strain CR1 possessed complete genetic repertoire (*bla*OXA-50 (B7D75_28665), *bla*PAO (B7D75_04145) genes with all the associated regulators and activators) required for β-lactam resistance providing evidence for pan -β-lactam resistance development in *P. aeruginosa* (Moya et al., 2012). Further, all outliers (*n* = 14) harbored *aph(3’)-IIb* (B7D75_04095) that confers resistance to several important aminoglycoside antibiotics, including kanamycin A and B, neomycin B and C, butirosin and seldomycin F5 (Zeng and Jin, 2003). The genes reported imparting chloramphenicol resistance were searched in the genome of CR1 strain. CR1 had *catA* gene (B7D75_19145) but had frameshift in the *catB7* gene (B7D75_21755), and *cmx* was exclusive to PA7 as a gene in RGP75 (Roy et al., 2010). *catB7* is an effector of chloramphenicol resistance (White et al., 1999). Only WH-SGI-V-07072 and WH-SGI-V-07370 along with PA7 showed the presence of *strA, strB, sul1* genes for aminoglycoside and sulphonamide resistance. Further, *aadB* (2″-aminoglycoside nucleotidyltransferase) was identified only in the genomes of WH-SGI-V-07072 and WH-SGI-V-07370 and was not present in any other genome including the complete genomes of CR1 and PA7.

PA7 has been exclusively known to possess amikacin resistance due to the presence of AAC4 gene and its product is an AAC (6′)-II constituted in the RGP75, but our analysis revealed that the gene is not exclusive to PA7 and these are also present in strain WH-SGI-V-07055. The genes exclusive to PA7 were a*ph(6)-Ic aph(3*′*)-IIa* in addition to *cmx* gene. Interestingly, strains CR1, ATCC9027, AZPAE14901, AZPAE15042, EML545, and EML548 lack the *arn*BCAD operon (present in PA7, PAO1 and UCBPP-PA14 and remaining outliers) required for resistance to polymyxin B and cationic antimicrobial peptides.

Point mutations in *gyrA* (Thr83Ile) and *parC* (Ser87Leu) known to be associated with Fluoroquinolone (FQ) resistance were checked by aligning both these genes among the 14 strains. Strains WH-SGI-V-07064, WH-SGI-V-07072 and the known multi-drug resistant strain PA7 had these mutations. Strain CR1 along with the remaining 10 strains showed susceptible genotype for Fluoroquinolones.

All the genomes had intact *mexABOprM* (B7D75_02135-02140-02145), *mexCDOprJ* (B7D75_23705-23700-23695), *mexEFOprN* (B7D75_12715-12710-12705) and its regulator *mexT* (B7D75_12720). CR1 had the *mexXYOprA* with *oprA* (B7D75_14890) gene linked to *mexXY* (B7D75_14880-14885) as in the genome of PA7. The multidrug efflux system was ubiquitously present in all the genomes. The analysis revealed that majority of outlier strains including CR1 possessed a reduced number of acquired resistant genes and is susceptible to a certain antibiotic(s), however, strains PA7, WH-SGI-V-07072, and WH-SGI-V-07370 were most pathogenic and resistant to majority of the antibiotics.

### Protein–Protein Interactions

**FIGURE 7:**
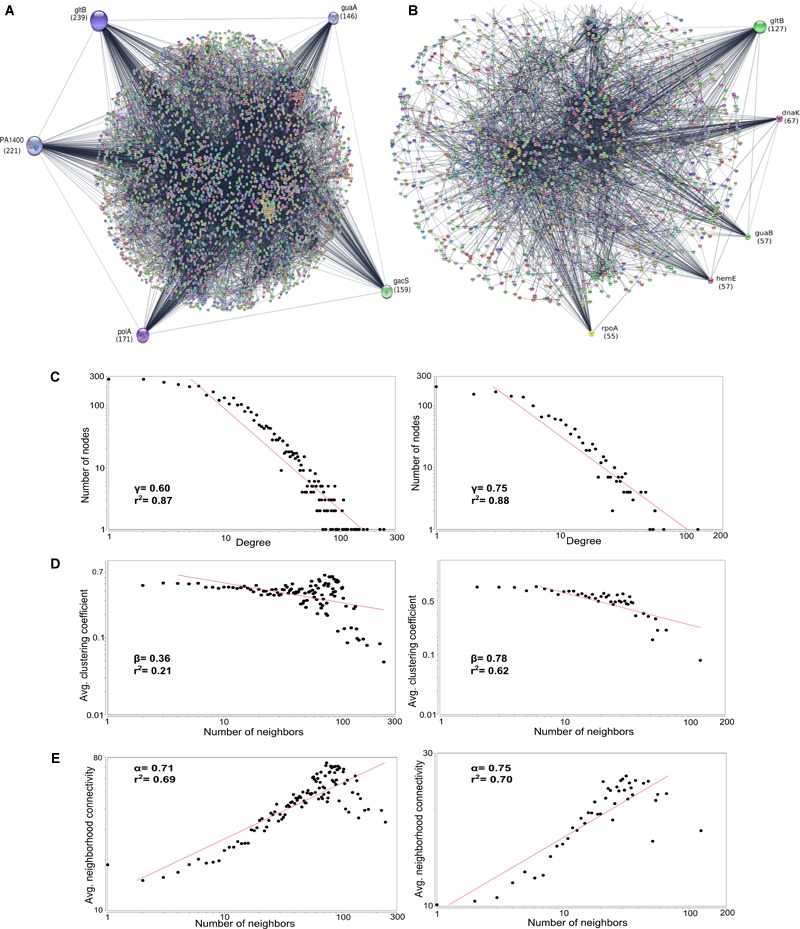
Protein–Protein Interaction network of **(A)** Core genes of outlier strains (*n* = 14) and **(B)** Core genes of classical strains (*n* = 64). The circles represent protein hubs and line represent edges. The radius of major regulatory hubs decreases as the number of interactions decreases. The topological properties of these networks depicting the correlation coefficient values (*r*^2^). **(C)** Node degree distribution, **(D)** average clustering coefficient, **(E)** average neighborhood connectivity. All these properties follow the power distribution and show the nature of the scale-free network and hierarchical organization.

The core amino acid sequences of the classical (*n* = 3199) and outlier (*n* = 4708) genomes were used for constructing complex PPI network using STRING Database v10.5 (Szklarczyk et al., 2017). The outlier strains showed five major hubs namely *gltB* (glutamate synthase), *PA1400* (pyruvate carboxylase), *polA* (DNA polymerase I), *gacS* (histidine kinase) and *guaA* (GMP synthase) whereas the classical strains had *gltB, dnaK* (chaperone protein), *guaB* (inosine 5′-monophosphate dehydrogenase), *hemE* (uroporphyrin decarboxylase) and *rpoA* (DNA-directed RNA polymerase) as the major regulatory hubs. *gltB* emerged to be the major regulator having the highest degree (*d*) in outliers (*d* = 239) as well as the classical strains (*d* = 127); whereas, rest of the regulatory hubs were unique for both the groups (Figure 7 and Table 5). The difference in the degree of association in both the groups can be attributed to higher overall nucleotide relatedness among outlier strains than between the members of the outlier and classical clades. Outliers had more genes in the core genome and therefore *gltB* had a higher degree of association in outlier PPI network. The gene *gltB* that codes for glutamate synthase [NADPH] large chain (EC 1.4.1.13), engaged in L-glutamate biosynthesis *via* GLT pathway (Pahel et al., 1978), which is itself part of amino acid biosynthesis has a role in the formation of enzymes involved in nitrogen assimilation (Janssen et al., 1981) and enhancing the yield of exotoxin A (Somerville et al., 1999). Further, two studies have shown that the *gltB* gene in concert with glutamine synthase *(glnA*) is a potential target for drug development (Mowbray et al., 2014; Murima et al., 2014). Interestingly, all the five hubs in case of outliers were connected to each other in sequential order according to the degree of interactions. However, in the classical strains, there was no interaction between *gltB* and the second prominent regulator *dnaK* which itself interacted with the next three hubs (Figure 7). Therefore, *gltB* can be regarded as the master of the proteome network with *dnaK* also playing an important role as it is the second most prominent regulatory hub (*d* = 67). Thus, targeting both these genes *gltB* and *dnaK*) can have a better impact against their pathogenesis (Chiappori et al., 2015). Therefore, in classical strains, a combination of drugs targeting both these regulatory hubs can prove to be more effective against multi-drug resistance. Similarly, the remaining major regulatory (Figure 7) protein hubs of the outliers can also be targeted as drug targets. Although these are considered as strains of the same species, the regulatory hub differs with *gltB* to be the major regulator.

**Table 5:** Major regulatory hubs from the core genome of an outlier and classical strains.

Regulatory hub	Degree	Protein	Function
**Outliers**			
*gltB*	239	Glutamate synthase	Provides glutamate for the glutamine synthetase reaction, absent in animals
*PA1400*	221	Pyruvate carboxylase	Irreversible carboxylation of pyruvate to form oxaloacetate (OAA)
*polA*	171	DNA polymerase I	Prokaryotic DNA replication
*gacS*	159	Histidine kinase	Play a role in signal transduction across the cellular membrane.
*guaA*	146	GMP synthase	Converts xanthosine monophosphate to guanosine monophosphate in the *de novo* synthesis of purine nucleotides,
**Classical**			
*gltB*	127	Glutamate synthase	Provides glutamate for the glutamine synthetase reaction, absent in animals
*dnaK*	67	Chaperone protein	DnaK is also involved in chromosomal DNA replication, possibly through an analogous interaction with the DnaA
*guaB*	57	Inosine 5′-monophosphate dehydrogenase	Purine biosynthetic enzyme; catalyzes the nicotinamide adenine dinucleotide (NAD+)-dependent oxidation of inosine monophosphate (IMP) to xanthosine monophosphate (XMP)
*hemE*	57	Uroporphyrin decarboxylase	Catalyzes the decarboxylation of four acetate groups of uroporphyrinogen-III to yield coproporphyrinogen-III
*rpoA*	55	DNA-directed RNA polymerase	Essential for life; significant role in transcription

*Hubs with their degree of association, the protein encoded and the function performed by these proteins. gltB was the major regulator in both the groups and four group specific regulatory hubs.*

The network followed a power scaling behavior as the degree exponent γ were 0.60 and 0.75, and the value was less than 2 (Barabasi and Oltvai, 2004) signifying the emergence of hierarchical modules and/or communities (Ravasz et al., 2002). Few highly connected hubs were connected to many low degree hubs indicating the regulatory power of the hubs over these nodes. Further, the average clustering coefficient and average neighborhood connectivity were calculated and it also followed power scaling law with β values of 0.36 and 0.78 and α values of 0.71 and 0.75 in outlier and classical strains, respectively, confirming that the network was hierarchical and the hub proteins in each network are indicative of the key molecules for habitat adaptation in each genome of the respective groups.

## Conclusion

Represented by very few genomic sequences, clinical strains of *P. aeruginosa* outlier group have shown to harbor novel virulence factors. We report here a non-clinical outlier strain CR1 characterized by a reduced genome size lacking few virulent factors found in clinical strains, the presence of a new type I secretion system from the non-*Pseudomonas* origin in RGP100 and a plasmid bearing trimeric autotransporter linked with VirB/D4 type IV secretion system. CR1 strain has genes responsible for siderophore production that is important in plants ability to acquire iron. We observed that outliers can be distinguished into 2 sub-clades one represented by CR1 having increased antibiotic susceptibility and other by strain PA7 having multidrug-resistant phenotypes. The genomes of the outliers were significantly more conserved than that of the classical clinical strains due to the high prevalence of core genes thus strengthening the fact that the two groups are significantly different. Outlier strains had genes normally found in soil-dwelling bacteria like ectoine degradation, plant-induced nitrilase in their pangenome that were absent in the classical strains. Although the outliers are considered as the strain of same species, they show major ambiguities in virulence factors, antibiotic resistance pattern, and protein regulatory hubs when compared with classical strains.

## Data Availability

The complete genome sequence of chromosome and plasmid of CR1 were deposited into the GenBank database with Accession number CP020560.1 and CP020561.2. The genome of CR1 was also submitted to PubMLST database with organism id 6661 for assigning new sequence type.

## Author Contributions

US, MS, and RL planned the study. DR provided the strain. US and PH did the experimental work for genomic DNA isolation and quantification. US, PH, RK, and AB did the computational analysis. All authors were involved in the writing and improving of the manuscript.

## Conflict of Interest Statement

The authors US, RK and RL were employed by the company PhiXGen Private Limited. The remaining authors declare that the research was conducted in the absence of any commercial or financial relationships that could be construed as a potential conflict of interest.

## Footnotes

1 https://github.com/widdowquinn/pyani

2 https://pubmlst.org/paeruginosa/

## Supplementary Material

FIGURE S1 3-D scatterplot of genome size (*x*-axis), number of coding sequences (*y*-axis) and DDH values (*z*-axis) among 14 outlier strains.

FIGURE S2 Heatmap with dual dendrogram based on the average nucleotide identity. The strains were compared using ANIm (mummer based) at default parameters. The matrix was then was plotted as a dual dendrogram in R (R Development Core Team, 2015).

FIGURE S3 Phylogeny based on 400 conserved marker genes using the maximum likelihood method. *Azotobacter vinelandii* **CA6** was taken as outgroup.

FIGURE S4 Phylogenetic tree of 78 *P. aeruginosa* genomes based on SNPs within the core genome was defined using Parsnp tool of the Harvest suite.

FIGURE S5 Circos representation of the whole genome alignment in strain CR1 and PA7 with a window size of 10 kb. Colors of arcs depict the orientation of the blocks (green: positive strand; red: negative strand).

FIGURE S6 Representation of new RGPs (RGP98-105) of CR1 against the type strain PA7. **(A)** RGP98, **(B)** RGP99, **(C)** RGP100, **(D)** RGP101, **(E)** RGP102, **(F)** RGP103, **(G)** RGP104, **(H)** RGP105.

Table S1 Soil properties of the isolation source of CR1 (rhizosphere soil of chili plantation).

Table S2 Details of all the 78 strains of *P. aeruginosa*.

Table S3 Annotation of environmental phage exclusive to CR1 as predicted by PHAST server.

Table S4 Complete list of regions of genomic plasticity in *P. aeruginosa*.

Table S5 Pathogenic potential of classical strains predicted by PathogenFinder.

FILE S1 Configuration file used in POTION pipeline.
