# Supplementary figures and images for: Comparative Genomic Analyses Reveal Core-Genome-Wide Genes Under Positive Selection and Major Regulatory Hubs in Outlier Strains of Pseudomonas aeruginosa
Source: Front Microbiol. 2019 Feb 6;10:53. doi: 10.3389/fmicb.2019.00053 (PMC6372532; doi:10.3389/fmicb.2019.00053)

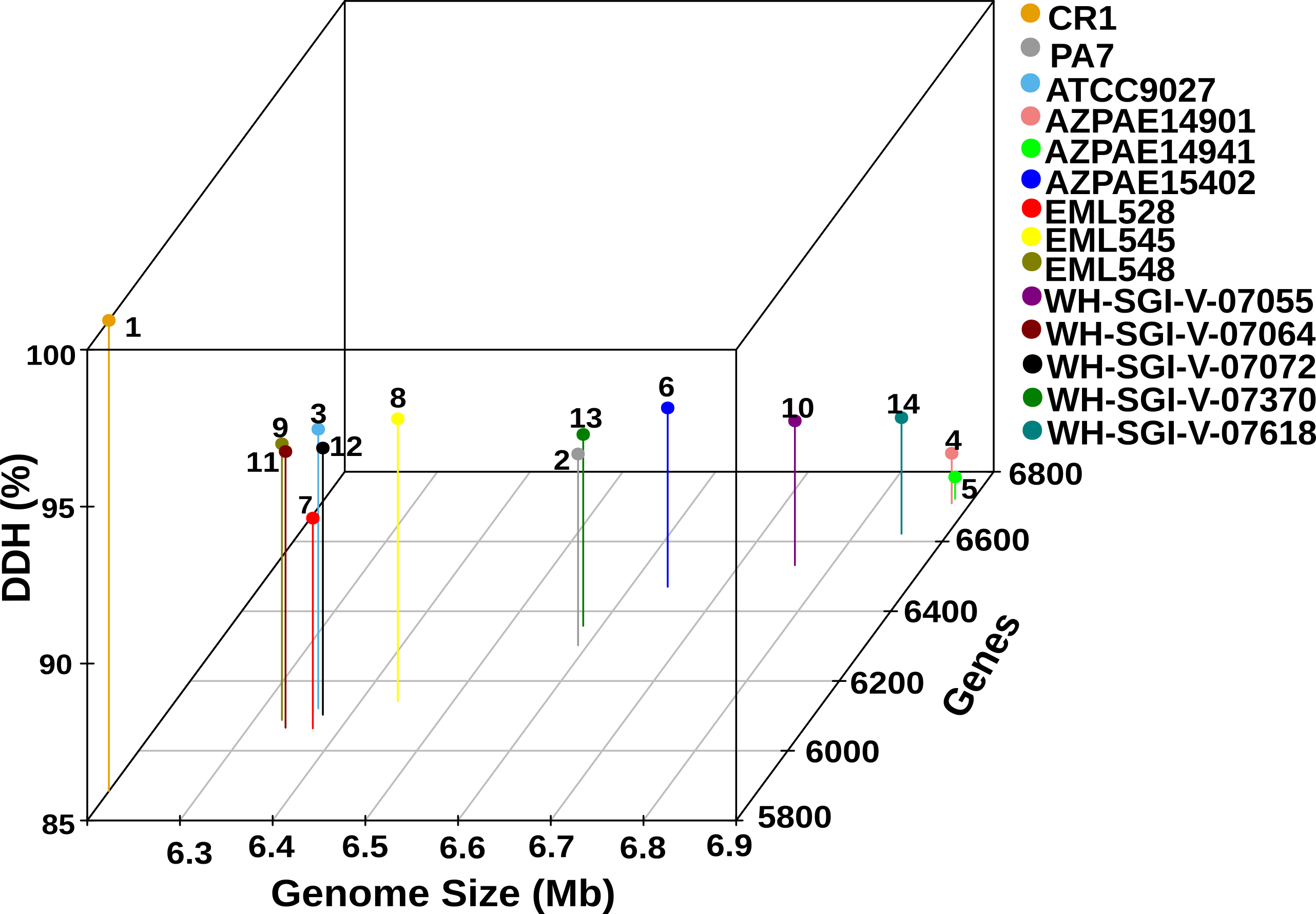

Supplement: FIGURE S1 — 3-D scatterplot of genome size (x-axis), number of coding sequences (y-axis) and DDH values (z-axis) among 14 outlier strains. [file Image_1.TIFF]

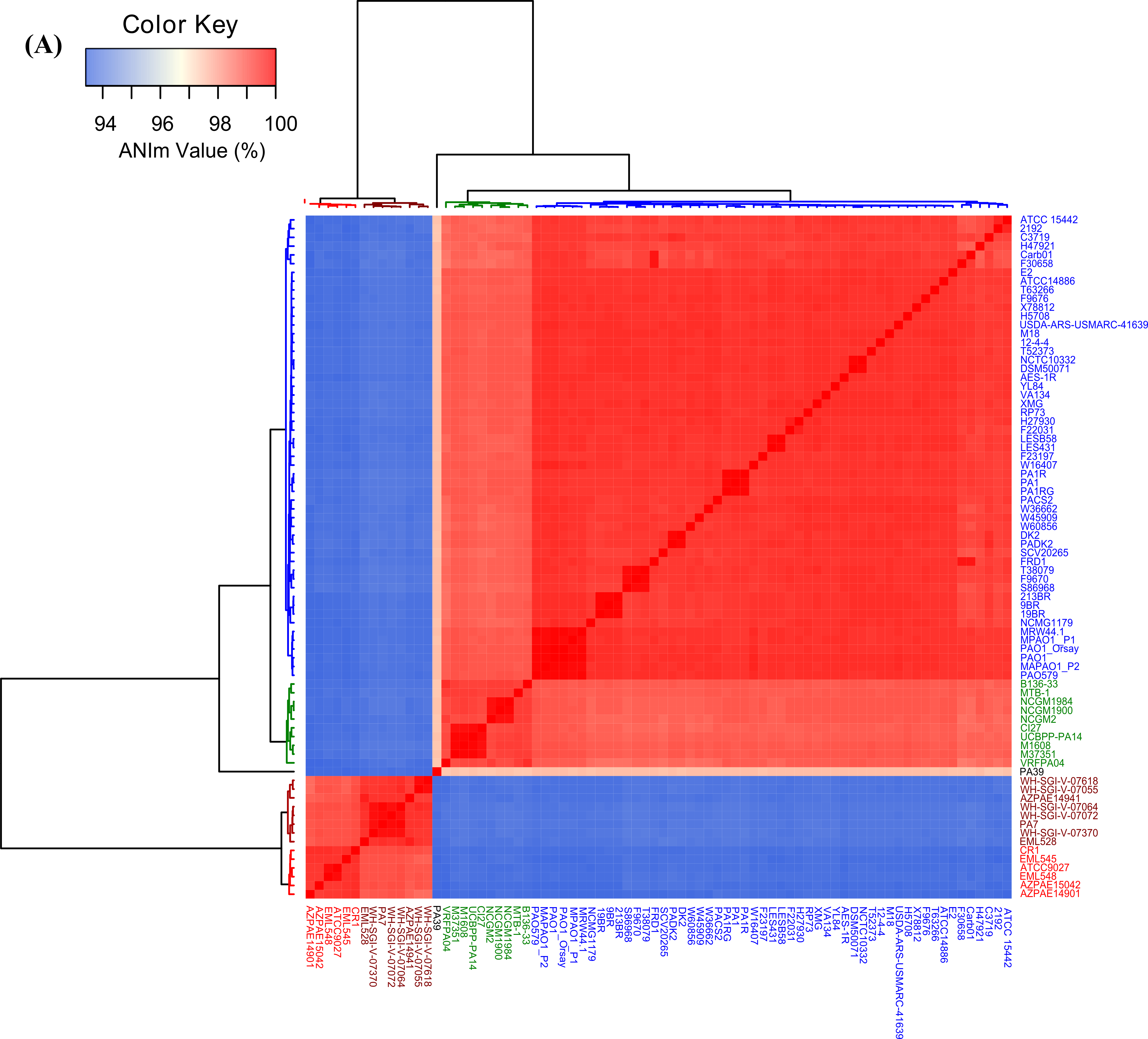

Supplement: FIGURE S2 — Heatmap with dual dendrogram based on the average nucleotide identity. The strains were compared using ANIm (mummer based) at default parameters. The matrix was then was plotted as a dual dendrogram in R (R Development Core Team, 2015). [file Image_2.TIFF]

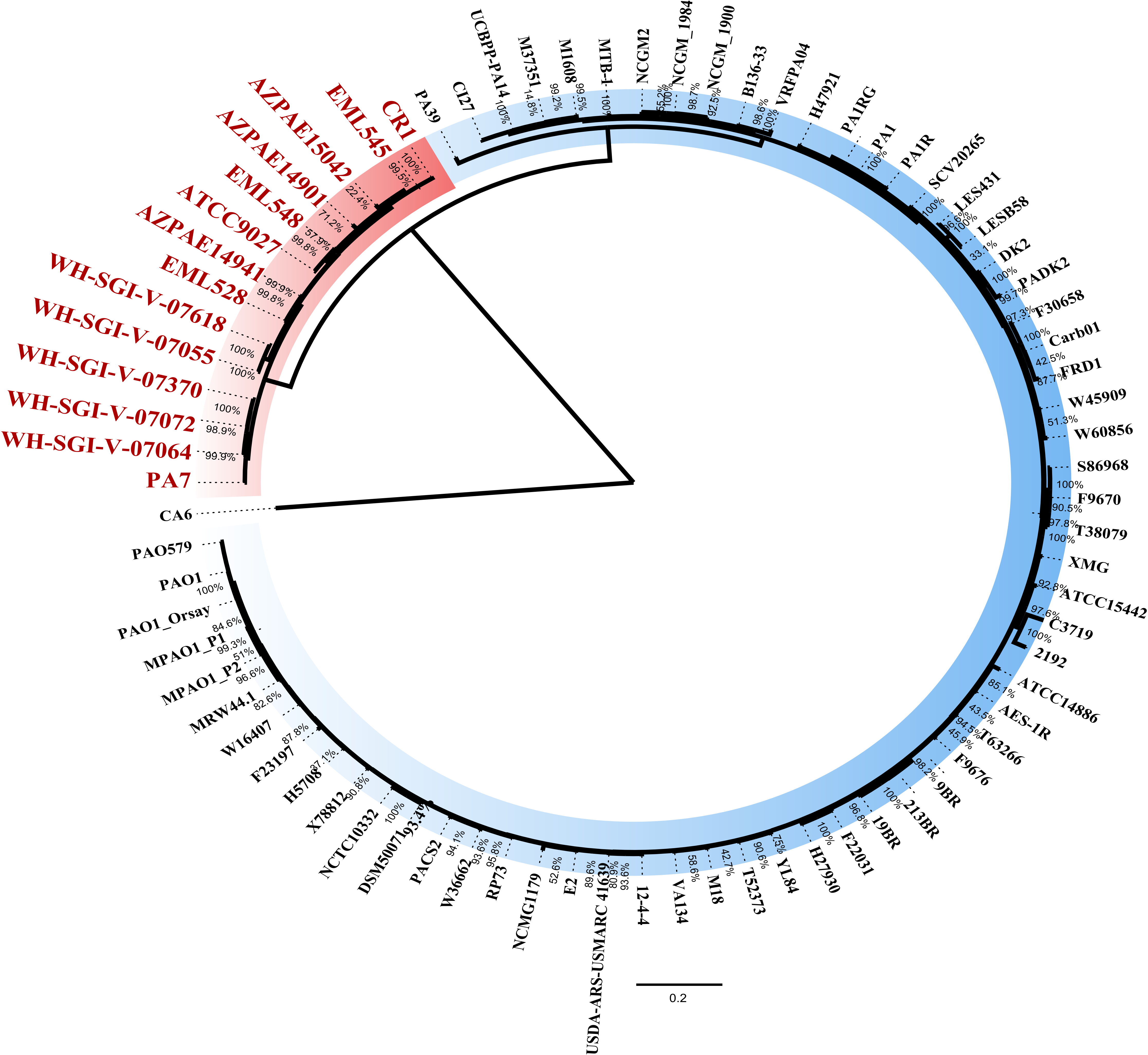

Supplement: FIGURE S3 — Phylogeny based on 400 conserved marker genes using the maximum likelihood method. Azotobacter vinelandii CA6 was taken as outgroup. [file Image_3.TIFF]

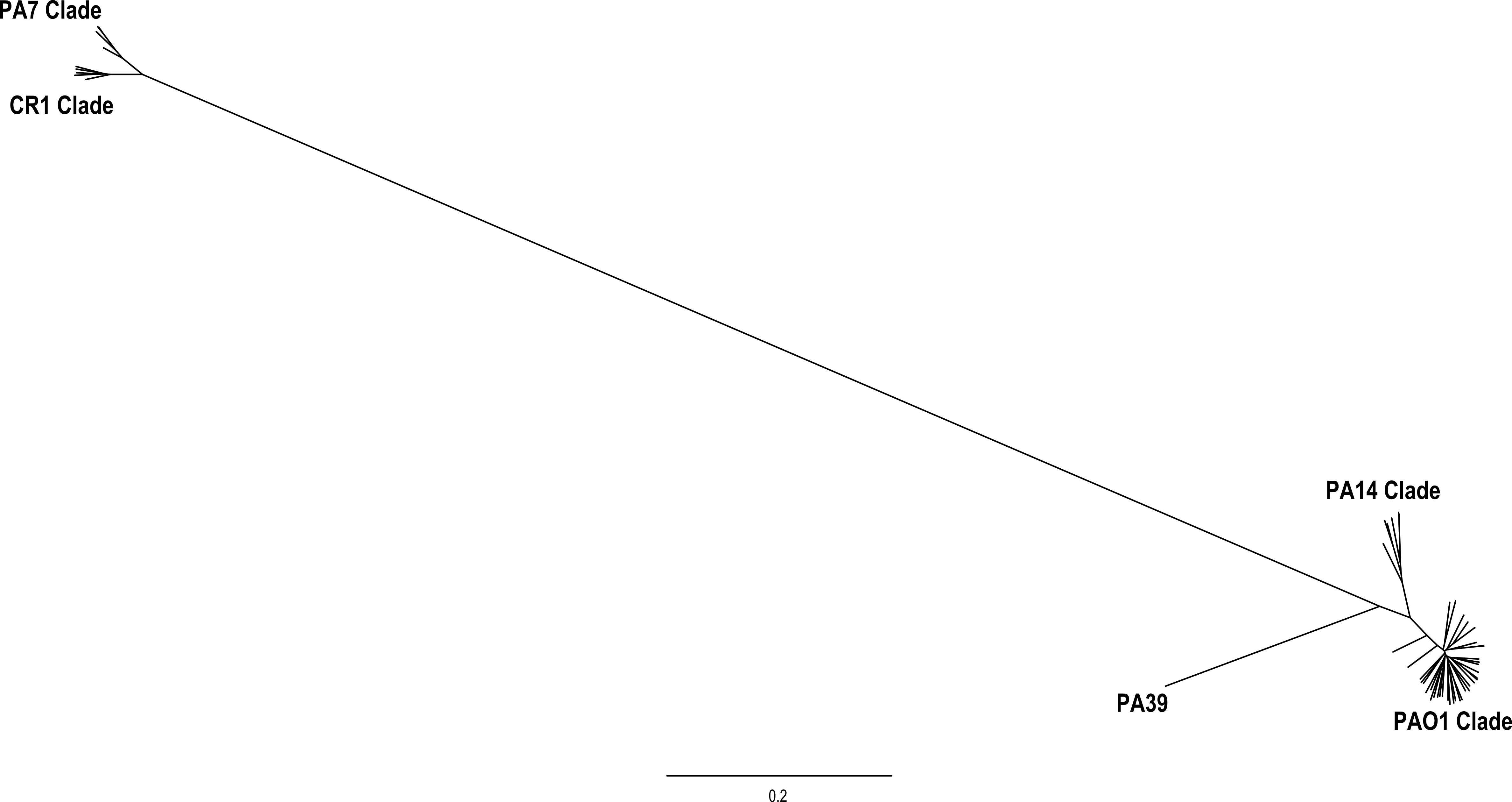

Supplement: FIGURE S4 — Phylogenetic tree of 78 P. aeruginosa genomes based on SNPs within the core genome was defined using Parsnp tool of the Harvest suite. [file Image_4.TIFF]

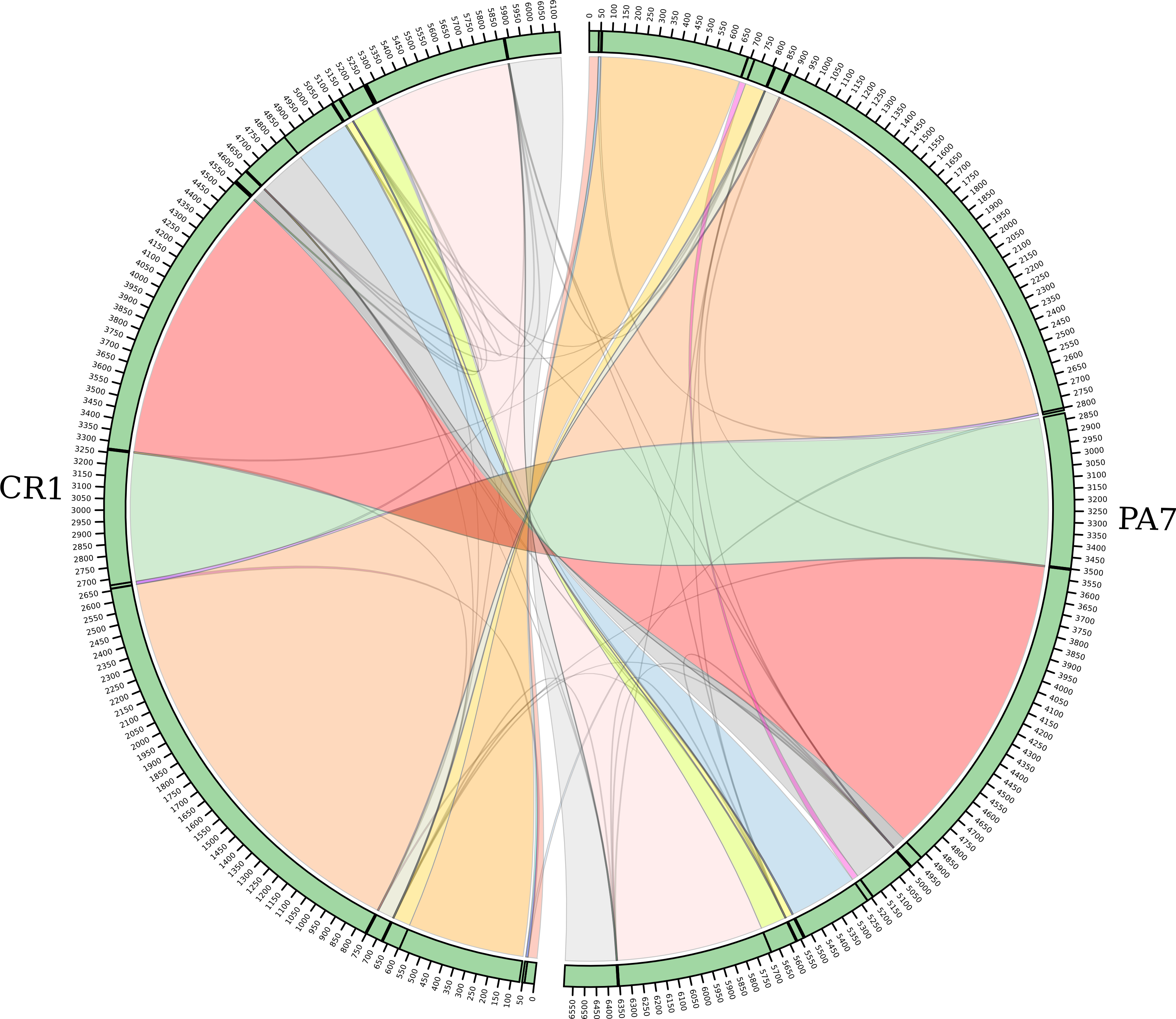

Supplement: FIGURE S5 — Circos representation of the whole genome alignment in strain CR1 and PA7 with a window size of 10 kb. Colors of arcs depict the orientation of the blocks (green: positive strand; red: negative strand). [file Image_5.TIFF]

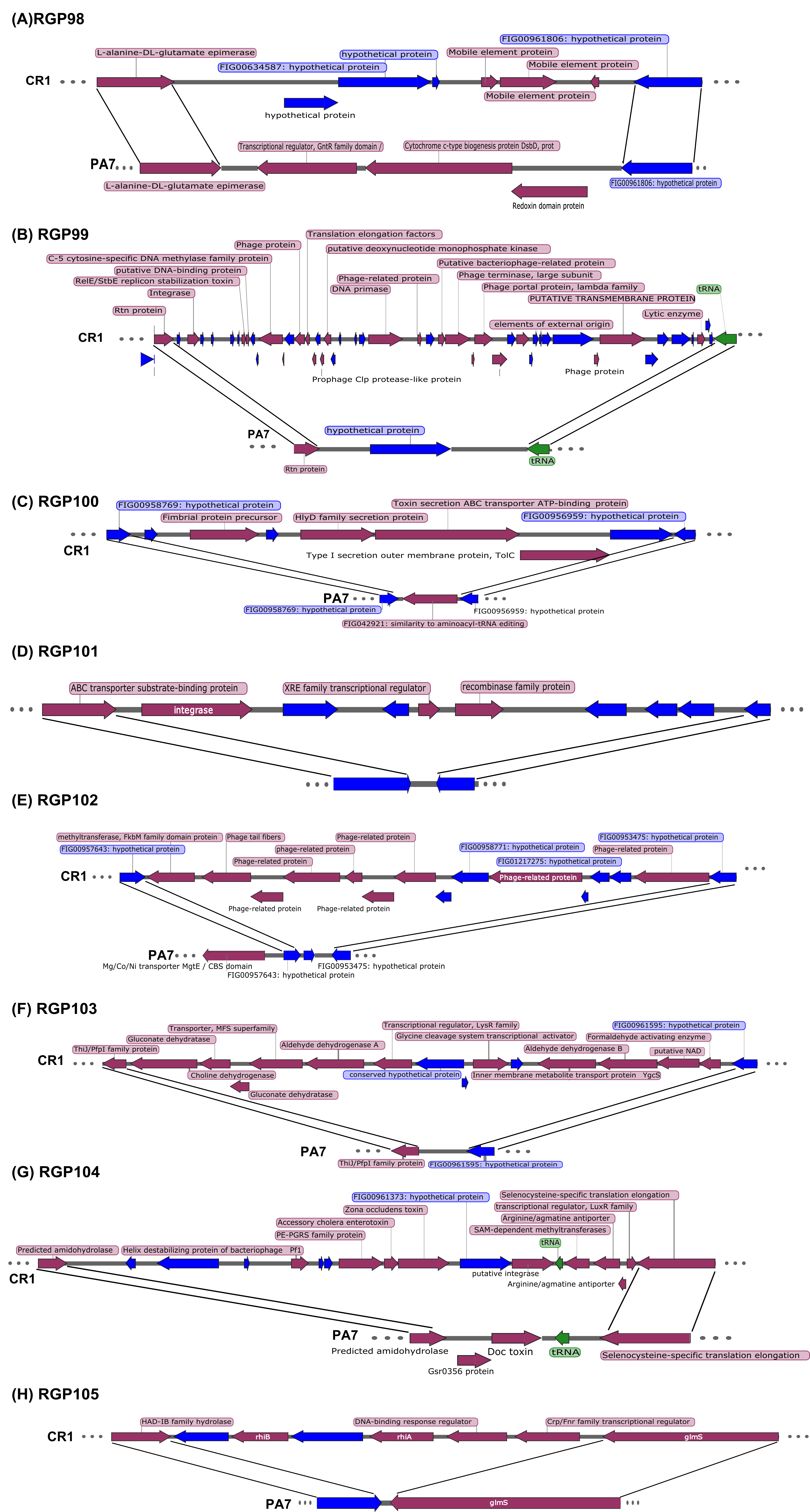

Supplement: FIGURE S6 — Representation of new RGPs (RGP98-105) of CR1 against the type strain PA7. (A) RGP98, (B) RGP99, (C) RGP100, (D) RGP101, (E) RGP102, (F) RGP103, (G) RGP104, (H) RGP105. [file Image_6.TIFF]
