# Supplementary material for: Comparative Genomic Analyses Reveal Core-Genome-Wide Genes Under Positive Selection and Major Regulatory Hubs in Outlier Strains of Pseudomonas aeruginosa
Source: Front Microbiol. 2019 Feb 6;10:53. doi: 10.3389/fmicb.2019.00053 (PMC6372532; doi:10.3389/fmicb.2019.00053)
Supplement: Table S1 — Soil properties of the isolation source of CR1 (rhizosphere soil of chili plantation). [file Table_1.DOCX]

| **Isolate** | **Location** | **GPS coordinate** | **Crop** | **Soil chemical Properties** | | | | | **Crop History** |
| --- | --- | --- | --- | --- | --- | --- | --- | --- | --- |
|  |  |  |  | **pH** | **Org C %** | **Av. N (kg/ha)** | **Av. P_2_O_5_ (kg/ha)** | **Av. K_2_O**  **(kg/ha)** |  |
| ***Pseudomonas aeruginosa* CR1** | Jonnalagadda,  Guntur Dt.  Andhra Pradesh, India | N= 16.10^0^N E= 28.29^0^E  31.5 m altitude | Chili | 6.71 | 0.42 | 302 | 31 | 824 | Chili grown with high chemical inputs |

**Supplementary Table 1: Soil properties of the isolation source of CR1 (Rhizosphere soil of chili plantation)**
