# Supplementary material for: Comparative Genomic Analyses Reveal Core-Genome-Wide Genes Under Positive Selection and Major Regulatory Hubs in Outlier Strains of Pseudomonas aeruginosa
Source: Front Microbiol. 2019 Feb 6;10:53. doi: 10.3389/fmicb.2019.00053 (PMC6372532; doi:10.3389/fmicb.2019.00053)
Supplement: Table S4 — Complete list of regions of genomic plasticity in P. aeruginosa. [file Table_4.DOCX]

**Supplementary Table 4:** Complete list of regions of genomic plasticity in *P. aeruginosa*

| **RGP No.** | **Insertion Site** | **Flanking loci PAO1 (PA_)** | **Flanking loci UCBPP-PA14 (PA14_)** | **Orfs included PA7 (PSPA7_)** | **Orfs included CR1 (B7D75_)** | **Features of interest encoded in CR1** |
| --- | --- | --- | --- | --- | --- | --- |
| RGP1 |  | 0201/0208 | 02530/02550 | 0285-0291 | 01025-01050 | ABC transporter |
| RGP2 | tRNA^Arg^ | 0256/0264 | 03160/03420 | Missing | Missing |  |
| RGP3 |  | 0611/0629 | 07960/08160 | 0756-0772 | 03085-03165 | phage related protein |
| RGP4 |  | 0641/0648 | 08300/08330 | 0785.1-0787.1 | Missing |  |
| RGP5 | tRNA^Gly^ | 0714/0730 | 55100/54830 | 4795-4797 | 21650-21730 | toxin/antitoxin, phage related protein |
| RGP6 | tmRNA | 0819/0827 | 53680/53560 | 4697-4700 | 21170-21180 | fimbrial protein and hypothetical proteins |
| RGP7 | tRNA^Lys^ | 0976/0988 | 51670/51510 | 4412-4530 | 20195-20285 | oxidoreductase, alcohol dehydrogenase, protease, TonB-receptor |
| RGP8 | tRNA^Ser^ | 1013/1014 | 51240/51220 | 4371-4382 | 19995-20050 | ectoine utilisation cluster |
| RGP9 |  | 1087/1092 | 50340/50290 | 4280-4289 | 19590-19610 | nucleotidyltransferase, hydrolase, methyltransferase |
| RGP10 |  | 1191/1192 | 49040/48870 | 4187 | 19145 | type A chloramphenicol O-acetyltransferase |
| RGP11 |  | 1222/1225 | 48520/48440 | 4163-4165 | 19030-19040 | dehydrogenase, LysR transcriptional regulator |
| RGP12 |  | 1243/1244 | 48160/48150 | 4148-4149 | 18960-18965 | *oprD* porin, hybrid sensor histidine kinase/response |
| RGP13 |  | 1367/1373 | 46630/46490 | 4011-4016 | 18310-18335 | heavy metal efflux |
| RGP14 |  | 1375/1376 | 46470/46540 | Missing | Missing |  |
| RGP15 |  | 1377/1394 | 46440/46390 | 3953-4007 | 18030-18290 | alpha/beta hydrolase, methyltransferase |
| RGP16 |  | 1530/1531 | 44650/44640 | Missing | Missing |  |
| RGP17 | tRNA^His^ | 1796/1797 | 41350/41280 | 3501-3502 | 16030-16035 | MipA/OmpV family protein, hypothetical |
| RGP19 |  | 1964/1965 | 39130/39110 | Missing | Missing |  |
| RGP20 |  | 2024/2070 | 38340/37730 | 3224-3263 | 14660-14850 | carbamoyl transferase, copper efflux, serine/ threonine transporter |
| RGP21 |  | 2099/2107 | 37360/37350 | Missing | Missing |  |
| RGP22 |  | 2181/2187 | 36370/36360 | 3114-3118 | 14135-14160 | manganese catalase, cytochrome oxidase, ferritin domain-containing protein |
| RGP23 |  | 2217/2235 | 36050/35690 | 3007-3071 | 13790-13925 | glycosylation enzymes, fimbrial proteins, the sensor kinase |
| RGP24 |  | 2422/2423 | 33370/33290 | 2834-2837 | 13100-13125 | CRISPRs with *cas* genes |
| RGP25 |  | 2455/2464 | 32860/32770 | 2775-2795 | 12870-12910 | haemagglutinins, hypothetical proteins |
| RGP26 | tRNA^Leu^ | 2570/2571 | 31290/30840 | 2648-2660 | 12225-12305 | integrases, hypothetical proteins |
| RGP27 | tRNA^Gly^ | 2583/2584 | 30700/30670 | 2617-2620 | 12050-12070 | transcriptional regulator, diguanylate phosphodiesterase |
| RGP28 | tRNA^Pro^ | 2727/2737 | 28895/28730 | 2513-2526 | 11560-11605 | group II intron reverse transcriptase, IS3 tranposase |
| RGP29 | tRNA^Gly^ | 2817/2820 | 27710/27590 | 2339 | 11055 | cyclic diguanylate phosphodiesterase |
| RGP30 |  | 2950/2951 | 25900/25880 | Missing | Missing |  |
| RGP31 |  | 3141/3160 | 23470/23360 | 1969-1986 | 09210-09315 | serotype O12 O-antigen locus |
| RGP32 |  | 3222/3223 | 22560/22490 | Missing | Missing |  |
| RGP33 |  | 3239/3240 | 22290/22075 | Missing | Missing |  |
| RGP34 |  | 3496/3515 | 18870/18860 | Missing | Missing |  |
| RGP35 |  | 3536/3537 | 18620/18610 | Missing | Missing |  |
| RGP36 |  | 3768/3769 | 15670/15340 | Missing | Missing |  |
| RGP37 |  | 3865/3870 | 13990/13850 | Missing | Missing |  |
| RGP38 |  | 4162/4163 | 10130/10040 | 0928-0932 | 03905-03920 | alcohol dehydrogenase, nuclear transport factor, transcriptional regulator |
| RGP39 |  | 4190/4196 | 09700/09690 | Missing | Missing |  |
| RGP41 | tRNA^Lys^ | 4541/4542 | 58900/60190 | Missing | Missing |  |
| RGP42 | tRNA^Met^ | 4673/4674 | 61820/61840 | 5324-5377 | 24080-24200 | lipase, chaperone, transcriptional regulator |
| RGP43 |  | 2770/2773 | 28280/28220 | 2460-2464 | 11315-11335 | N-acetyltransferase, sensor domain-containing diguanylate cyclase |
| RGP44 |  | 4100/4108 | 10850/10820 | 0988-0995 | 04165-04200 | two-component sensor histidine kinase |
| RGP46 |  | 0041/0042 | 00510/00530 | 0043-0046.1 | 00215-00275 | haemagglutinins, tranposase |
| RGP47 |  | 1149/1153 | 49530/49500 | 4228-4230 | 19340 | phage antirepressor |
| RGP48 |  | 1238/1242 | 48240/48170 | Missing | Missing |  |
| RGP50 |  | 1655/1656 | 43110/43050 | Missing | Missing |  |
| RGP52 |  | 1934/1940 | 39500/39460 | 3353 | 15270 | hypothetical protein |
| RGP53 |  | 2332/2337 | 34450/34440 | Missing | Missing |  |
| RGP56 |  | 2793/2795 | 28000/27980 | 2363-2463 | 11175-11325 | phage proteins, chemotaxis protein, glutamate carboxypeptidase |
| RGP58 | tRNA^Arg^ | 3366/3368 | 20560/20490 | Missing | 08160-08185 | type VI secretion system proteins |
| RGP60 | tRNA^Thr^ | 4524/4526 | 58700/58750 | 5143-5161 | 23295-23305 | phage related protein |
| RGP62 | tRNA^Phe^ | 5149/5150 | 68000/68040 | Missing | Missing |  |
| RGP63 |  | 0069^ | 00810^ | 0070-0139 | 00395^ | radical SAM protein |
| RGP64 |  | 0278^ | 03620^ | 0355-0368 | 01360^ | sulfite exporter TauE/SafE family protein |
| RGP65 |  | 0377/0378 | 04940/04950 | 0473-0476 | 01865-01890 | IS3 transposase, hydrolase |
| RGP66 | tRNA^Met^ | 0574/0575 | 07450/07500 | 0678-0716 | 02895 | bifunctional diguanylate cyclase/phosphodiesterase |
| RGP67 |  | 3858/3859 | 14080/14100 | 1247-1252 | 05450-05500 |  |
| RGP68 |  | 3840/3844 | 14290/14340 | 1268-1272 | 05580-05600 | type VI secretion system proteins |
| RGP69 |  | 3714/3715 | 16340/16350 | 1407-1420 | 06510-06575 | type II secretion pathway cluster |
| RGP70 | tRNA^Pro^ | 3031/3032 | 24860/24880 | 2108-2125 | 09915-010000 | multidrug transporter, exporters, NRPS genes |
| RGP71 |  | 2650/2651 | 29820/29830 | 2550-2555 | 11725-11750 | vacuolar transporter, porin. transcriptional regulator |
| RGP72 | tRNA^Cys^ | 2581/2582 | 30710/30730 | 2622-2633 | 12080-12150 | gluconolaconase, oxidoreductase, galactonate dehydatase, aldolase |
| RGP73 |  | 2397/2403 | 33600/33690 | 2858-2861 | 13230-13240 | NRPS, hydrolase, siderophore receptor |
| RGP74 |  | 2201/2202 | 36230/36250 | 3089-3094 | 14005-14045 | iron transport |
| RGP75 |  | 1579/1580 | 44070/44080 | 3695-3747 | Missing |  |
| RGP76 |  | 1425/1428 | 45980/46010 | 3902-3912 | 17780-17830 | alginate export family protein, monooxygenase, dioxygenase |
| RGP77 |  | 1397/1398 | 46330/46350 | 3943-3949 | 17980-18010 | lipase, hypothetical proteins, VgrG protein |
| RGP78 |  | 4466/4467 | 57990/57990 | 5040-5080 | Missing |  |
| RGP79 |  | 5290/5291 | 69840/69850 | 6033-6063 | Missing |  |
| RGP80 |  | 5454/5460 | 72000/72060 | 6245-6257 | 28310-28375 | phosphate metabolism |
| RGP81 |  | 4138/4139 | 10420/10380 | Missing | Missing |  |
| RGP82 |  | 3663/3664 | 16980/16970 | Missing | Missing |  |
| RGP83 |  | 3463/3464 | 19330/19320 | Missing | Missing |  |
| RGP84 | tRNA^Ser^ | 2603/2604 | 30430/30410 | Missing | Missing |  |
| RGP85 |  | 2593/2594 | 30550/30560 | Missing | Missing |  |
| RGP86 |  | 0831/0832 | 53510/53520 | Missing | Missing |  |
| RGP87 | tRNA^Thr^ | 5160/5161 | 68140/68170 | Missing | Missing |  |
| RGP88 |  | 3961~ | 12630~ | Missing | Missing |  |
| RGP89 |  | 3834/3836 | 14440/14390 | 1278 | 05630 | hypothetical protein |
| RGP90 |  | 2533/2536 |  | Missing- | 12495-12500 | transcriptional regulator, oxidoreductase |
| RGP91 |  | 2545/2548 |  | 2690-2691 | 12435-12440 | transcriptional protein, hypothetical protein |
| RGP92 |  |  |  | 2635 | 12605-12610 | fimbrial protein, transcriptional regulator |
| RGP93 |  | 2588/2591 |  | Missing | Missing |  |
| RGP94 | tRNA^Ser^ | 2593/2604 |  | Missing | Missing |  |
| RGP95 |  | 3326/3337 |  | Missing | Missing |  |
| RGP96 |  | 3361/3363 |  | 1768 | 08210 | hypothetical protein |
| RGP97 |  | 3386/3388 |  | Missing | Missing |  |
| RGP98 |  | 4040/4041 | 11580/11590 | 1062-1064 | 04510-04545 | mobile genetic elements, helicase, partitioning proteins |
| RGP99 | tRNA^Leu^ | 3824/3825 | 14550/14570 | Missing | 05680-05935 | phage proteins |
| RGP100 |  | 1840/1842 | 40690/40710 | 3451 | 15760-15785 | type I secretion system proteins |
| RGP101 |  | 1530/1531 | 46640/46650 | Missing | 17255-17295 | hypothetical protein |
| RGP102 |  | 0910/0911 | 52480/42490 | Missing | 20645-20705 | hypothetical protein |
| RGP103 |  | 4336/4337 | 56380/56390 | Missing | 22280-22335 | formaldehyde activating enzyme, aldehyde dehydrogenase, aldo/keto reductase, gluconate dehydratase |
| RGP104 | tRNA^Ser^ | 4802/4802.1 | 63440/63460 | 5523-5524 | 24905-24995 | amino acid permease, arginine/agmatine antiporter, SAM dependent methyltransferase |
| RGP105 |  | 5547/5549 | 73150/73170 | Missing | 28835-28860 | *rhiA, rhiB* genes, transcriptional regulators |
