# Supplementary material for: Comparative Genomic Analyses Reveal Core-Genome-Wide Genes Under Positive Selection and Major Regulatory Hubs in Outlier Strains of Pseudomonas aeruginosa
Source: Front Microbiol. 2019 Feb 6;10:53. doi: 10.3389/fmicb.2019.00053 (PMC6372532; doi:10.3389/fmicb.2019.00053)
Supplement: FILE S1 — Configuration file used in POTION pipeline. [file Data_Sheet_1.PDF]

```
###############PROJECT PARAMETERS#######################

mode = site                              # main analysis mode. Currently
POTION supports only site-models analysis.

CDS_dir_path = /home/ruplal/Desktop/software/CDS_outliers_RAST/

homology_file_path = /home/ruplal/Desktop/software/POTION-
1.1.2/outlier_groups

project_dir_path = /home/ruplal/Desktop/software/POTION-
1.1.2/result_outlier

max_processors = 100

remove_identical = yes ###############PROJECT
PARAMETERS#######################

mode = site                              # main analysis mode. Currently
POTION supports only site-models analysis.

CDS_dir_path = /home/ruplal/Desktop/software/CDS_outliers_RAST/

homology_file_path = /home/ruplal/Desktop/software/POTION-
1.1.2/outlier_groups

project_dir_path = /home/ruplal/Desktop/software/POTION-
1.1.2/result_outlier

max_processors = 100

remove_identical = yes                 # "yes" to remove 100% identical
nucleotide groups at the very beginning of
                                         # analysis, "no" otherwise

verbose = 1                              # 1 to print nice log messages
telling you what is going on. 0 otherwise

############SEQUENCE/GROUP PARAMETERS################

groups_to_process = all                  # Defines which lines of the
cluster file (ortholog groups) will be processed.
                                         # Use "all" to process every
group, "-" to set groups between two given lines
                                         # (including the said lines).
                                         # Use "!" to not process a
specific line, can be used with "-" to specify a
                                         # set to not be processed.
Useful if groups are taking too long to finish.
                                                    # Use "," or ";" to
set distinct sets
                                                    # Examples: 1;4-10;12
will process groups 1, 4 to 10 and group 12
                                                    #           all;!3
will process all groups, except group 3
                                                    #           all;!3-5
will process all groups, except groups 3 to 5
```

```
behavior_about_bad_clusters = 1          # what should POTION do if it
finds a cluster with a sequence removed
                                         # due to any filter? Possible
options are:
                                         # 0 - does not filter any
sequence (not recommended)
                                         # 1 - removal of any flagged
sequence
                                         # 2 - removal of any group with
flagged sequences

homology_filter = 1              # this variable controls for what POTION
will do if a group with paralogous
                                         # genes is found. Possible
options are:
                                         # 0 - analyze all sequences
within group
                                         # 1 - remove all paralogous
within group, analyzing only single-copy genes
                                         # 2 - remove groups with
paralogous genes
                                         # 3 - remove single-copy genes,
analyzing all paralogous within group together
                                         # 4 - remove single-copy genes
and split remaining paralogous into individual
                                         # species, evaluating each
subgroup individually

validation_criteria = all                # quality criteria to remove
sequences. Possible values are:
                                         # 1 - checks for valid start
codons
                                         # 2 - checks for valid stop
codons
                                         # 3 - checks for sequence size
multiple of 3
                                         # 4 - checks for nucleotides
outside ATCG
                                         # 'all' applies every
verification

additional_start_codons = ()             # these codons, plus the ones
specified in codon table, will be the valid start
                                         # codons for validation purposes
additional_stop_codons = ()              # same as start codons

codon_table = 11

absolute_min_sequence_size = 150         # minimum sequence length cutoff
for sequence/group further evaluation

absolute_max_sequence_size = 10000       # maximum sequence length cutoff
for sequence/group further evaluation

relative_min_sequence_size = 0.83        # sequences smaller than
mean|meadian times this value will be filtered
```

```
relative_max_sequence_size = 1.2         # sequences greater than
mean|meadian times this value will be filtered

sequence_size_average_metric = mean      # which average metric will be
calculated to determine the
                                         # minimum/maximum relative
lengths ranges for sequence removal
                                         # Possible values are "mean" and
"median"


min_group_identity = 70                 # mean minimum group identity
cutoff in pairwise sequence alignments

max_group_identity = 100                # mean maximum group identity
cutoff in pairwise sequence alignments

group_identity_comparison = aa          # the kind of sequence that will
be used when computing mean group identity
                                        # possible values are "nt" or
"aa"

min_sequence_identity = 70               # minimum (mean/median) sequence
identity cutoff in pairwise sequence alignments

max_sequence_identity = 100             # maximum (mean/median) sequence
identity cutoff in pairwise sequence alignemnts

sequence_identity_average_metric = mean # would you like to use mean or
median to measure sequence identity?
                                        # possible values are "mean" and
"median"

sequence_identity_comparison = aa       # the kind of sequence that will
be used when computing sequence identity
                                        # possible values are "nt" and
"aa"

min_gene_number_per_cluster = 3         # minimum # genes in group after
all filtering steps

max_gene_number_per_cluster = 14        # maximum # genes in group after
all filtering steps

min_specie_number_per_cluster = 3       # minimum # species in group
after all filtering steps

max_specie_number_per_cluster = 14      # maximum # species in group
after all filtering steps

reference_genome_file =  PA7   # genome reference name, leave blank for
none (same name used in fasta file)


###########THIRD-PARTY SOFTWARE CONFIGURATION###############

multiple_alignment = prank              # program used for multiple
sequence alignment. Possible values are
```

```
                                        # muscle, mafft and prank

bootstrap = 100                          # number of bootstraps in
phylogenetic analysis

phylogenetic_tree_speed = fast           # fast or slow analysis? Used in
phylip dnaml or proml only

phylogenetic_tree = dnaml               # program used for phylogenetic
tree reconstruction. Possible values are
                                        # proml dnaml, phyml_aa and
phyml_nt

recombination_qvalue = 0.1              # q-value for recombination
detection. Must occur for all the specified tests,
                                        # or 0 | N.A. to skip
recombination test

rec_minimum_confirmations = 2            # minimum number of significant
recombination tests positives (1-3), or N.A. to
                                         # skip recombination test

rec_mandatory_tests = phi                # any combination of the three
test names, separated by spaces, or N.A. to use
                                         # any test

remove_gaps = strict                     # numeric values between 0 and 1
will remove columns with that percentage of
                                         # gaps. Values of "strict" or
"strictplus" will use respectively these
                                         # filters to remove unreliable
regions (described in trimal article)

PAML_models = m12 m78                    # codeml models to be generated.
"m12" and/or "m78" values acceptable.
pvalue = 0.05                            # p-values for positive
selection detection
qvalue = 0.05                            # q-values for positive
selection detection
```
